# Arabidopsis root defense barriers support beneficial interactions with rhizobacterium *Pseudomonas simiae* WCS417

**DOI:** 10.1111/nph.70549

**Published:** 2025-09-04

**Authors:** Jiayu Zhou, Melissa Uribe Acosta, Max J. J. Stassen, Run Qi, Ronnie de Jonge, Fred White, Gertjan Kramer, Lemeng Dong, Corné M. J. Pieterse, Ioannis A. Stringlis

**Affiliations:** ^1^ Plant‐Microbe Interactions, Department of Biology Science4Life, Utrecht University P.O. Box 800.56 3508 TB Utrecht the Netherlands; ^2^ Institute of Botany Jiangsu Province and Chinese Academy of Sciences 210014 Nanjing China; ^3^ AI Technology for Life, Department of Information and Computing Sciences Science4Life, Utrecht University 3584 CH Utrecht the Netherlands; ^4^ Swammerdam Institute for Life Sciences, University of Amsterdam 1098 XH Amsterdam the Netherlands; ^5^ Laboratory of Plant Pathology Agricultural University of Athens 75 Iera Odos st. 11855 Athens Greece

**Keywords:** *Arabidopsis thaliana*, beneficial rhizobacterium, camalexin, plant growth promotion, reactive oxygen species, root colonization, root defense barriers

## Abstract

- Plant roots interact with pathogenic and beneficial microbes in the soil. While root defense barriers block pathogens, their roles in facilitating beneficial plant–microbe associations are understudied. Here, we examined the impact of specific root defense barriers on the well‐known beneficial association between *Arabidopsis thaliana* and the plant growth‐promoting rhizobacterium *Pseudomonas simiae* WCS417.
- Using 15 Arabidopsis mutants with alterations in structural (cutin, suberin, callose, and lignin) and chemical (camalexin and glucosinolates) defense barriers, we demonstrate that some barriers impact WCS417‐mediated plant growth responses and its root colonization.
- Root exudates from Arabidopsis wild‐type (WT) and mutant plants differentially affected the WCS417 transcriptome, with camalexin notably impacting bacterial motility and chemotaxis, which was also confirmed by *in vitro* studies. On the plant side, WCS417‐induced transcriptome changes in the roots of defense barrier mutants were significantly different from those in WT plants, particularly affecting growth and defense‐related processes. Specifically, the data indicated altered activity of reactive oxygen species in several of the defense barrier mutants, which was confirmed *in planta*.
- Our data suggest that various root defense barriers play a role in balancing growth and defense during this mutualistic interaction, thereby impacting the establishment and effectiveness of plant mutualists, extending their established role in disease resistance.

## Introduction

Plants interact with many microbes dwelling on and inside their roots, collectively known as root microbiota (Berendsen *et al*., 2012; Bai *et al*., 2022). These microbes can be neutral or beneficial, while some can cause diseases. Plants secrete nearly 30% of their photosynthates into the rhizosphere (Paterson & Sim, 2000), thereby providing nutrients to root microbiota. Root exudates can not only nurture microbes but also exert a selective pressure, creating a gradient of decreased microbial diversity but increased abundance of selected microbial taxa in the interphase between bulk soil and root surface, known as the rhizosphere effect (Bakker *et al*., 2018). Root exudates contain both nutrients and selective compounds that largely shape the microbial community in the rhizosphere (Bais *et al*., 2006). Primary metabolites released in the rhizosphere nurture the rhizosphere microbial community, favoring microbes with a high relative growth rate (López *et al*., 2023).

Root‐derived secondary metabolites, such as coumarins (Stringlis *et al*., 2018b; Voges *et al*., 2019), triterpenes (Huang *et al*., 2019), glucosinolates (Bressan *et al*., 2009), and flavonoids (Kudjordjie *et al*., 2021), can inhibit specific microbiota while having little influence on others (Stringlis *et al*., 2018b). For example, triterpenes directly promote the growth of Proteobacteria but inhibit Actinobacteria (Huang *et al*., 2019). Additionally, *Azospirillum* and *Flavobacterium* are enriched in the roots of Arabidopsis glucosinolate and flavonoid mutants (Kudjordjie *et al*., 2021). Besides the chemical defense barriers mentioned above, plants can select root‐associated microbes through structural defense barriers. Such barriers are the Casparian strip and suberin, which can alter the diffusion properties of primary and secondary metabolites in roots and consequently affect the composition of the root‐associated microbiome (Durr *et al*., 2021; Salas‐González *et al*., 2021).

Most studies so far have focused on the roles of chemical and structural defense barriers in the interaction between plants and pathogens (De Coninck *et al*., 2015; Pascale *et al*., 2020; Fröschel *et al*., 2021), demonstrating their roles as defense components. For instance, pathogen infection of Arabidopsis roots or stimulation of the roots by the flagellin epitope flg22 leads to accumulation of chemical defense components, such as glucosinolates (Ludwig‐Müller *et al*., 1999) or camalexin, one of the most well‐studied phytoalexins (Bednarek *et al*., 2005; Millet *et al*., 2010). Additionally, structural defense components function as barriers in root pathogen interactions, such as ligno‐suberin (Fröschel *et al*., 2021; Kashyap *et al*., 2022), cutin (Chassot *et al*., 2007), lignin (Chezem *et al*., 2017), and callose (Millet *et al*., 2010). Although the roles of chemical and structural root defense barriers in restricting pathogen colonization have been extensively studied, their functions in the interplay between plants and beneficial microbes remain largely unexplored (Pascale *et al*., 2020). Here, we use the well‐established beneficial interaction between *Arabidopsis thaliana* (hereafter: Arabidopsis) and *Pseudomonas simiae* WCS417 (hereafter: WCS417) to examine our hypothesis that dysfunctions of chemical and structural defense barriers can affect the outcomes of plant–microbe interactions.

WCS417 promotes plant growth, enhances root development, induces systemic resistance, and improves abiotic stress tolerance (Pieterse *et al*., 2021). Several important features of WCS417 have been identified with a role in this beneficial plant–microbe interaction (Pieterse *et al*., 2021). To achieve root colonization, WCS417 suppresses local host defense responses in the roots that are otherwise activated by its microbe‐associated molecular patterns (MAMPs; Millet *et al*., 2010; Stringlis *et al*., 2018a; Yu *et al*., 2019). Furthermore, WCS417 induces the biosynthesis and exudation of coumarins in Arabidopsis roots (Stringlis *et al*., 2018b), which in turn modulate WCS417 motility and enhance root colonization (Yu *et al*., 2021). WCS417 also increases suberin deposition in the endodermis of Arabidopsis roots (Verbon *et al*., 2023). Besides coumarins and suberin, we expect that other chemical or structural defense barriers in roots might play a role in the beneficial interplay between Arabidopsis and WCS417. To systematically study the roles of chemical and structural defense barriers, we investigated an array of plant responses to WCS417 root colonization in 15 different Arabidopsis mutants affected in cutin, suberin, Casparian strip, lignin, callose, camalexin, or glucosinolates. By analyzing rhizobacteria‐mediated plant growth promotion, root development, root colonization, and WCS417 and Arabidopsis root transcriptome profiles, we gained novel insights into the role of these root defense barriers in this beneficial plant–microbe interaction.

## Materials and Methods

### Plant materials and growth condition

In this study, *Arabidopsis thaliana* (L.) Heynh. accession Columbia‐0 (Col‐0) and different root defense barrier mutants were used, including cutin‐biosynthesis or deficient mutants *bdg‐1*, *dcr‐2*, and *gpat4/8* (Berhin *et al*., 2019); suberin and Casparian strip mutants *esb1*, *myb36‐2/sgn3‐3*, and *sgn3‐3* (Salas‐González *et al*., 2021); lignification mutants *myb15‐1* and *myb15‐2* (Chezem *et al*., 2017); callose deposition mutant *pmr4‐1* (Millet *et al*., 2010); camalexin production mutant *pad3‐1* (Zhou *et al*., 1999); and glucosinolate deficient mutants *myb28/28*, *myb51*, *myb34/51/122*, *cyp79b2/b3*, and *gtr1/2* (Beekwilder *et al*., 2008; Frerigmann & Gigolashvili, 2014; Frerigmann *et al*., 2016; Xu *et al*., 2017). For all genotypes, seeds were surface sterilized for 4 h with chlorine gas as described (Van Wees *et al*., 2013) and then sown on agar‐solidified 1× Murashige and Skoog (MS) medium supplemented with 0.5% sucrose (Murashige & Skoog, 1962). After 2 d of stratification at 4°C, the Petri dishes were transferred to a 10 h : 14 h, light : dark growth chamber at 22°C (light density 100 μmol m^−2^ s^−1^) and positioned vertically (Stringlis *et al*., 2018b).

### Rhizobacteria cultivation

*Pseudomonas simiae* strain WCS417 was cultivated on King's medium B (KB) agar plates supplemented with 50 μg ml^−1^ rifampicin at 28°C. After overnight cultivation, bacterial cells were collected from the plate and washed twice in 10 mM MgSO_4_, with centrifugation in between for 5 min at 4500 ***g***. The bacterial cells were then suspended in 10 mM MgSO_4_ to an optical density at 600 nm (OD_600_) of 0.1, representing 10^8^ CFU ml^−1^ (Verbon *et al*., 2023).

### Plant growth and root development parameter measurement and data processing

Seven‐day‐old uniform seedlings of all the genotypes were transplanted from 1× MS medium with 0.5% sucrose to agar‐solidified Hoagland medium without sucrose (2 mM Ca(NO_3_)_2_, 5 mM KNO_3_, 2 mM MgSO_4_, 2.5 mM KH_2_PO_4_, 70 μM H_3_BO_3_, 14 μM MnCl_2_, 1 μM ZnSO_4_, 0.5 μM CuSO_4_, 10 μM NaCl, 0.2 μM Na_2_MoO_4_, 50 μM Fe(III)‐ ethylenediaminetetraacetic acid, 4.7 mM MES, and 0.75% agar, pH = 5.5) mixed with the stock WCS417 culture (10^8^ CFU ml^−1^) to a final density of 10^5^ CFU ml^−1^ according to the method described by Herrera Paredes *et al*. (2018). Control seedlings were transplanted to Hoagland medium mixed with an equal volume of 10 mM MgSO_4_. Seedlings were harvested 7 d after transplanting and plant growth parameters (shoot fresh weight, primary root length, and lateral root number) were measured as described by Stringlis *et al*. (2018a).

The relative shoot fresh weight, primary root length, and lateral root number of each genotype were calculated by dividing the values for each WCS417‐treated seedling by the average value of mock‐treated seedlings. When the relative shoot weight, primary root length, or lateral root number of a genotype is one, WCS417 has no influence on the shoot growth or root architecture of that genotype. When the relative values are higher or lower than one, WCS417 has a positive or negative effect, respectively, on these plant growth parameters.

The average shoot fresh weight, primary root length, and lateral root number of all seedlings per plate were calculated across treatments and genotypes and used as variables in a principal component analysis (PCA), providing an integrated visualization of how treatments and genotypes affected Arabidopsis phenotypes. To statistically assess the influence of treatment, genotype, and their interaction on plant phenotypes, a permutational multivariate analysis of variance (PERMANOVA) was performed based on the shoot fresh weight, primary root length, and lateral root number of individual seedlings.

### Bacterial colonization

Arabidopsis roots were detached using a sterile knife and harvested, while agar *c*. 5 cm from the roots was collected in the same plate. Both root and agar samples were weighed and placed in a sterile tube with 1 ml of 10 mM MgSO_4_ and four glass beads. Root and agar samples were homogenized in a TissueLyser II (Qiagen) for 2 min at 30 oscillations s^−1^. These samples were then serially diluted using 10 mM MgSO_4_, plated onto KB‐agar plates supplemented with 50 μg ml^−1^ rifampicin, and incubated at 28°C for 24 h. After incubation, bacterial colony‐forming unit (CFU) numbers per gram of harvested roots or agar were counted and calculated as described (Herrera Paredes *et al*., 2018).

### Root exudate collection

Root exudates from the Arabidopsis genotypes were collected according to Yu *et al*. (2021). In brief, 7‐d‐old uniform seedlings were transferred from 1× MS medium with 0.5% sucrose to agar‐solidified Hoagland medium without sucrose. They were then cultured for seven more days under the same growth conditions. When 14 d old, the seedlings were rinsed three times in Milli‐Q water and transferred to ECO boxes containing 25 ml Milli‐Q water. After transferring 100 seedlings in each ECO box, the boxes were placed back in the climate chamber. After 3 d, root exudates from each ECO box were collected by filtering the content over 0.2 μm Millipore filters (Merck KGaA, Darmstadt, Germany), after which the exudates were stored at −80°C until further use.

### Bacterial growth in root exudates

To determine the effect of different root exudates on bacterial growth, WCS417 was inoculated in 96‐well microtiter plates, each well containing 200 μl of undiluted root exudates mixed with a bacterial suspension. The final concentration of WCS417 in root exudates was 10^4^ CFU ml^−1^ (OD_600_ = 0.00001). The 96‐well microtiter plates with bacteria were wrapped in aluminum foil and incubated in the climate chamber. Bacterial CFUs were assessed at 24 h after inoculation by plating the dilution series of bacterial cultures on KB‐agar plates supplemented with 50 μg ml^−1^ rifampicin. The plates were incubated at 28°C for 24 h, after which the bacterial CFU numbers were counted.

Bacterial samples for RNA sequencing were treated with root exudates and collected according to Yu *et al*. (2021). In brief, WCS417 cells were inoculated from a bacterial stock at 10^10^ CFU ml^−1^ in 50‐ml Falcon tubes, each containing 10 ml of undiluted root exudate, to a bacterial density of OD600 = 0.15. Tubes with bacteria were incubated in a shaker at 100 rpm at 21°C in the dark. After 1 h of incubation in the root exudates, 1 ml of bacterial culture was taken and mixed with 2 ml of RNAprotect® Bacteria Reagent (Qiagen) by vortexing for 5 s, after which they were incubated for 5 min at Room Temperature according to the manufacturer's instructions. The bacterial suspension was then pelleted by centrifugation at 5000 ***g*** for 10 min and stored at −80°C until RNA extraction.

### Bacterial RNA extraction and cDNA library preparation

Total bacterial RNA was extracted using the RNeasy Mini Kit (Qiagen). Lysozyme (Sigma‐Aldrich Inc., St Louis, MO, USA) was used in the enzymatic lysis step, and QIAGEN RNase‐Free DNase Set (Qiagen) was used in the on‐column DNase digestion step, according to the instructions in the RNAprotect® Bacteria Reagent Handbook. The quality of total RNA was analyzed using the Agilent RNA6000 Nano Kit (Agilent Technologies, Waldbronn, Germany).

Ribosomal RNA depletion and library preparation were conducted using the Zymo‐Seq RiboFree® Protocol (Zymo Research, Irvine, CA, USA) with 500 ng of total RNA. The quality of the cDNA library was measured with the Agilent TapeStation System (Agilent Technologies, Waldbronn, Germany).

### Bacterial RNA sequencing and data analysis

Libraries from all the samples were pooled for sequencing in the same flow cell of an Illumina® NextSeq500 platform in a single‐end run with a read length of 75 bp (Illumina, San Diego, CA, USA). FastQC was used to check the read quality. Kallisto was used to quantify the abundance of transcripts (Bray *et al*., 2016) based on the coding sequences of WCS417 (Berendsen *et al*., 2015) retrieved from the National Center for Biotechnology Information (https://www.ncbi.nlm.nih.gov). From the Kallisto output, the estimated counts were used in the differential gene expression analysis, which was performed using the DESeq2 package in R (Love *et al*., 2014). There were 5502 WCS417 genes detected with at least 10 read counts in at least one sample (Supporting Information Table S1). The estimated counts of each gene were normalized using variance stabilizing transformation (VST). Then, the influences of Milli‐Q water and different root exudates on the gene expression patterns of WCS417 were analyzed using PCA. Subsequently, a PERMANOVA was performed to statistically assess the influence of these treatments on bacterial gene expression.

Genes with a log_2_ fold change ≥ 1 or ≤ −1 and a false discovery rate (FDR) < 0.05 were selected as differentially expressed genes (DEGs). To visualize the expression patterns of DEGs across treatments, a heatmap was generated using normalized expression values, and *k*‐means clustering was applied to group DEGs with similar expression patterns. Briefly, *k*‐means is an unsupervised and iterative clustering algorithm that partitions genes into *k* clusters based on the similarity of their expression patterns. The algorithm initializes *k* cluster centroids randomly and assigns each gene to the nearest centroid according to Euclidean distance. The centroids are subsequently updated as the mean expression profile of all genes within each cluster, and this process iterates until convergence. Following clustering, genes were reordered in the heatmap based on their cluster assignments, allowing clearer visualization of distinct expression patterns across treatments. Enrichment analysis of DEGs within each cluster was performed using the clusterProfiler package in R (Yu *et al*., 2012) to identify overrepresented biological pathways and functional categories.

### Bacterial motility and chemotaxis assay

For the bacterial motility assay, WCS417 was transferred from KB‐agar medium using a sterile toothpick to Cook's cytophaga semi‐solidified (soft) medium (CCM, 0.2% bacto tryptone and 0.3% agar in distilled water) supplemented with 1% (v/v) dimethyl sulfoxide (DMSO) or 1.0 mM camalexin (Sigma‐Aldrich), 1% (v/v) as described (Yu *et al*., 2021). Either camalexin or DMSO was uniformly mixed into the agar‐solidified medium. The agar was gently pierced by the toothpicks with bacteria to ensure that the toothpicks did not touch the plate bottom. Plates were inoculated overnight at 28°C. The diameter of the bacterial colony was then measured. To determine whether the effects of camalexin on bacterial motility are general or specific, three additional bacterial strains were assessed using the method described above, including *Pseudomonas syringae* pv *tomato* (*Pst*) DC3000, *Rhizobium* sp. YAF28, and *Bacillus subtilis* GB03. Among these, *Pst* DC3000 is a well‐known plant pathogenic bacterium, while the others were isolated from the Arabidopsis rhizosphere and represent different genera.

The bacterial chemotaxis assay was conducted according to Reyes‐Darias *et al*. (2016) with some modifications. A volume of 200 μl of 1.0 mM camalexin in 1% DMSO, 10 mM MgSO_4_ or 1% DMSO, and 10 mM MgSO_4_ as a control was drawn into a syringe with a needle. The needle was placed in a 250‐μl bacterial suspension in 10 mM MgSO_4_ (OD_600_ = 0.1) for 30 min. Then, the camalexin and control solutions in the syringes were flushed out, serially diluted, and dropped on KB plates supplemented with 50 μg ml rifampicin. After incubation at 28°C for 24 h, the CFU numbers of WCS417 that had entered the syringe were counted and calculated.

### Bacterial root colonization of Arabidopsis grown in a soil–sand mixture

Soil experiment is conducted according to the method described by Van Wees *et al*. (2013). In detail, *c*. 9 kg of soil (Proveen, Bas Van Buuren, the Netherlands) was mixed with 4 kg of river sand and sterilized by double autoclaving. Half of the sterilized mixture (*c*. 6.5 kg) was inoculated with 500 ml of WCS417 suspension at a final density of 10^6^ CFU g^−1^ soil, while the other half was mixed with 500 ml of 10 mM MgSO_4_ as a mock. Seeds of Arabidopsis Col‐0 and *pad3‐1* were surface sterilized and germinated on 1× MS medium with 0.5% sucrose as described before. Eleven‐day‐old uniform seedlings were transplanted into the WCS417‐ or mock‐treated soil–sand mixture. The seedlings grew for another 14 d under short‐day conditions (22°C; 10 h : 14 h, light : dark; light density 100 μmol m^−2^ s^−1^) before harvest. To assess WCS417 root colonization, Arabidopsis roots were gently shaken to remove adhering soil, and bacterial number on Col‐0 and *pad3‐1* roots was quantified as described before.

### Untargeted metabolite profiling of root exudates

Root exudates previously collected from different genotypes were freeze‐dried and resuspended in 1 ml of 100% methanol. The solutions were sonicated for 5 min, vortexed thoroughly, and centrifuged at maximum speed to obtain the supernatant. Methanol was subsequently evaporated from the supernatant using a speed vacuum concentrator, and the dried extracts were resuspended in 150 μl of 20% methanol. Quality control samples were generated by pooling 10 μl from each individual sample to create a mixture, which was then divided into three aliquots for analysis.

Samples were analyzed by untargeted liquid chromatography‐mass spectrometry profiling, using a Quadrupole Time‐of‐Flight Mass Spectrometer equipped with a dual‐stage trapped ion mobility separation cell (timsTOF Pro; Bruker Daltonics, Billerica, MA, USA). The injection volume was 10 μl. Chromatographic separation was performed on an Ultimate RS UHPLC system (Thermo Scientific, Germeringen, Germany) equipped with an Acquity UPLC CSH C18 column. A gradient elution was applied, increasing from 1 to 99% acetonitrile over 18 min at a flow rate of 0.4 ml min^−1^. The mobile phases consisted of 0.1% formic acid in water (Solvent A) and acetonitrile (Solvent B). Eluting compounds were ionized in both positive and negative ion modes using an Apollo II ion funnel electrospray ionization (ESI) source. The source parameters were as follows: capillary voltage set to +4500 V or − 3500 V, end plate offset at 500 V, drying temperature at 250°C, desolvation gas flow at 8 l min^−1^, and nebulizer gas pressure at 3.0 bar. Auto MS/MS acquisition was conducted with a switching threshold of 350 counts, a cycle time of 0.5 s, and a collision energy range of 20–30 eV.

### Data analysis of untargeted metabolite profiling of root exudates

Data were processed using MetaboScape v.5.0 for peak deconvolution and alignment. Peaks were recalibrated using internal lock masses. Metabolic features were annotated with the SMARTFORMULA algorithm and several spectral libraries, including Bruker MetaboBASE v.3.0, Bruker HDBM v.2.0, MetaboBASE v.2.0 (in silico), MS‐DIAL LipidDBs, MoNA VF NPL QTOF, and an in‐house Arabidopsis metabolite library. Feature tables were exported for downstream statistical analysis (Table S2).

After correcting for technical blanks and no‐plant controls, stringent filtering was applied based on the experimental design. Features were retained only if they were present in all replicates of at least one genotype and detected in a minimum of two genotypes overall. This filtering step resulted in a set of 2223 features for subsequent analysis. Partial least squares discriminant analysis (PLS‐DA) was conducted to show genotype‐associated differences in metabolite profiles. Subsequently, a PERMANOVA was performed to statistically assess the influence of genotype on metabolite composition. In addition, a hierarchical clustering analysis was used to visualize similarities in exudation patterns among genotypes.

### Root colonization by WCS417 and sample collection for RNA extraction

To test the effect of WCS417 on the root transcriptome in different Arabidopsis genotypes, 9‐d‐old uniform seedlings of each genotype were transferred from 1× MS medium with 0.5% sucrose to 6‐well plates (two seedlings per well) with each well containing 2 ml of liquid 1× MS medium with 0.5% sucrose (Stringlis *et al*., 2018a). Subsequently, the seedlings were cultured for seven more days under the same growth conditions. The day before treatment with WCS417, the medium in each well was replaced with 2 ml of fresh 1× MS medium with 0.5% sucrose. When the seedlings were 17‐d‐old, 20 μl of a WCS417 bacterial suspension (10^10^ CFU ml^−1^ MgSO_4_) or an equal volume of 10 mM MgSO_4_ was added to each well. Six hours after treatment, the roots of the seedlings were collected. For each genotype and treatment, four biological replicate samples were taken, each consisting of 12 pooled, similarly treated root systems, which were immediately snap‐frozen in liquid nitrogen and stored at −80°C.

### Plant RNA extraction and cDNA library preparation

Arabidopsis roots were homogenized in a TissueLyser II for 2 min at a frequency of 30 oscillations per second. Subsequently, RNA was extracted using the RNeasy Mini Kit according to the manufacturer's instructions. Lysozyme was used in the enzymatic lysis step, and the QIAGEN RNase‐Free DNase Set was used in the on‐column DNase digestion step. The quality of total RNA was analyzed using an Agilent TapeStation System and quantified using a Qubit™ 2.0 RNA HS (High Sensitivity) Assay Kit (Thermo Fisher, Waltham, MA, USA).

Paramagnetic beads coupled with oligo d(T)25 were used to isolate poly(A) + mRNA from total RNA according to the instructions in the NEBNext® Poly(A) mRNA Magnetic Isolation Module manual (New England BioLabs Inc., Ipswich, MA, USA). Before first strand cDNA synthesis, mRNA samples were randomly primed (5′ d(N6) 3′ [N = A,C,G,T]) and fragmented based on the manufacturer's recommendations (New England BioLabs Inc.). The first strand was synthesized with Protoscript II Reverse Transcriptase with an extension period of 30 min at 42°C. All remaining steps for library construction were used according to the NEBNext® Ultra™ II Non‐Directional RNA Library Prep Kit for Illumina® (New England BioLabs Inc.). Final library quantity was assessed using Qubit™ 2.0 and quality was assessed using the Agilent High Sensitivity D1000 ScreenTape System (Agilent Technologies Inc., Santa Clara, CA, USA). The final average library size was *c*. 400 bp with an average insert size of *c*. 250 bp.

### Plant RNA sequencing and data analysis

Equimolar pooling of libraries was performed based on Quality Control values and sequenced on an Illumina® NovaSeq platform (Illumina) in a paired‐end run with a read length of 150 bp (20 million paired‐end reads per sample with 10 million in each direction). The read length was then trimmed to 2 × 75 bp. FastQC was used to check the read quality. Kallisto was used to quantify the abundance of transcripts (Bray *et al*., 2016) based on the Arabidopsis transcriptome reference from Ensembl Plant Database (plants.ensembl.org). From the Kallisto output, the estimated counts were used in the differential analysis, which was performed using the DESeq2 package in R (Love *et al*., 2014). In the Arabidopsis transcriptome dataset, 21 501 genes were detected with at least 10 read counts in at least one sample (Table S3). Subsequently, read counts for each gene across samples were normalized using VST. The impacts of WCS417 treatment and genotype on Arabidopsis root gene expression patterns were visualized by PCA. Subsequently, a PERMANOVA was performed to statistically assess the influence of WCS417 treatment and genotype on gene expression. Genes with a log_2_ fold change ≥ 1.5 or ≤ −1.5 and an FDR < 0.01 were selected as DEGs. DEGs with similar expression patterns were grouped by *k*‐means clustering and visualized using a heatmap. Enrichment analysis of DEGs within each cluster was performed using the clusterProfiler package in R (Yu *et al*., 2012) to identify overrepresented biological pathways and functional categories. The overlap of DEGs across genotypes was shown using an UpSet plot.

### ROS production measurement

ROS production in Arabidopsis roots was measured basically according to Bisceglia *et al*. (2015). In brief, 7‐d‐old uniform seedlings were transferred from 1× MS medium with 0.5% sucrose to 6‐well plates (two seedlings per well) containing 2‐ml liquid 1× MS medium with 0.5% sucrose. They were then cultured for seven more days under the same growth conditions. The day before treatment with flg22 or WCS417, the roots were detached, placed in six‐well plates, and immersed in Milli‐Q water. The plates were covered with aluminum foil overnight to eliminate the probable ROS production caused by cutting (Bisceglia *et al*., 2015). The roots then were dried with absorbent paper and transferred into 96‐well microtiter plates. Each well contained two roots in 200 μl reaction mixture (30 μM L‐012 and 5 × 10^−4^ U μl^−1^ horseradish peroxidase diluted in Milli‐Q water) and either WCS417 bacteria at final densities of 10^5^ or 10^8^ CFU ml^−1^ or 0.2 μM flg22. Luminescence in each well was recorded on a Glomax luminometer (Promega Corp., Madison, WI, USA) at 2.5‐min intervals. Log_2_‐transformed relative luminescence units were used for subsequent data analysis.

### Data analysis

Statistic data analysis is conducted in R (v.4.0.5).

## Results

### Root defense barriers affect WCS417‐mediated shoot growth promotion and root architecture changes

**Fig. 1:**
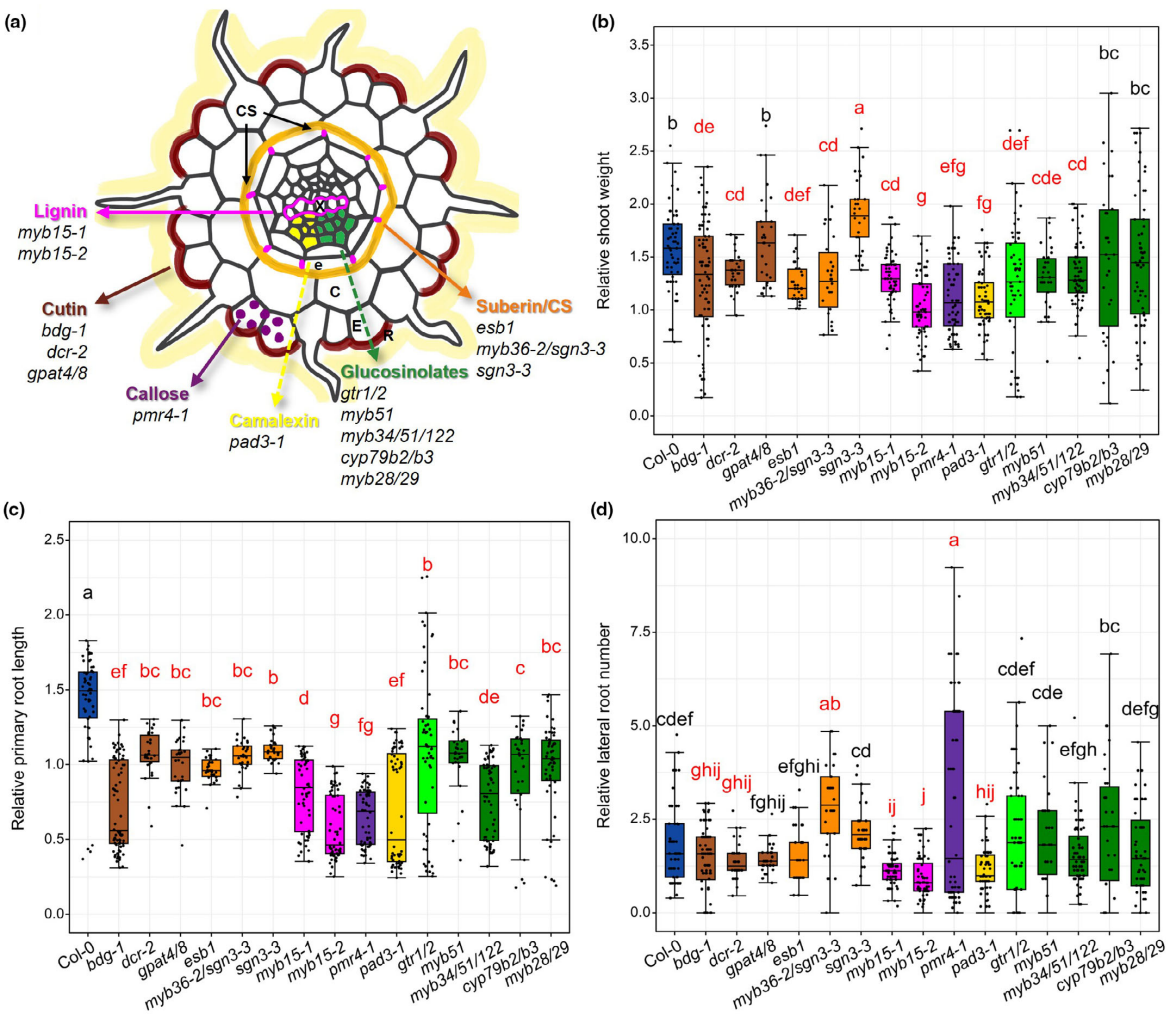
Root defense barrier mutants display different shoot growth and root architecture changes in response to *Pseudomonas simiae* WCS417. (a) Schematic cross‐section of *Arabidopsis thaliana* root showing the localization and activity of structural and chemical defense components in different cell layers and corresponding mutants described previously (Zhou *et al*., 1999; Beekwilder *et al*., 2008; Millet *et al*., 2010; Frerigmann & Gigolashvili, 2014; Frerigmann *et al*., 2016; Chezem *et al*., 2017; Xu *et al*., 2017; Berhin *et al*., 2019; Salas‐González *et al*., 2021). C, cortex; CS, casparian strip; e, endodermis; E, epidermis; R, rhizosphere; X, xylem. Dashed arrows represent chemical defense components exuded into the rhizosphere; solid arrows represent structural defense components. Relative shoot fresh weight (b), primary root length (c), and lateral root number (d) of Arabidopsis Columbia‐0 (Col‐0) and defense barrier mutants at 7 d after transplanting seedlings to Hoagland medium without sucrose containing 10^5^ CFU ml^−1^ WCS417. Relative shoot fresh weight for each seedling was calculated by dividing the shoot weight of each WCS417‐treated seedling by the average shoot weight of mock‐treated seedlings. Relative primary root length and lateral root number were calculated in the same way. In the boxplots, the horizontal line inside each box represents the median; the top and bottom edges of each box represent the 75^th^ and 25^th^ quartiles, respectively; and the upper and lower whiskers extend to 1.5× the interquartile range from the top and bottom of the box, respectively. Different lowercase letters represent significant differences among indicated genotypes (one‐way ANOVA with least significant difference test, *P* < 0.05). Red lowercase letters indicate mutants that show significant differences compared with Col‐0. Different groups of defense barrier mutants are shown in colors matching the ones shown in (a). Each data point is a biological replicate, representing a single Arabidopsis seedling. The number of seedlings (*n*) per genotype and treatment is as follows: Col‐0 (Mock: *n* = 60; WCS417: *n* = 59), *bdg‐1* (Mock: *n* = 80; WCS417: *n* = 80), *dcr‐2* (Mock: *n* = 30; WCS417: *n* = 30), *gpat4/8* (Mock: *n* = 30; WCS417: *n* = 30), *esb1* (Mock: *n* = 30; WCS417: *n* = 30), *myb36‐2/sgn3‐3* (Mock: *n* = 30; WCS417: *n* = 30), *sgn3‐3* (Mock: *n* = 30; WCS417: *n* = 30), *myb15‐1* (Mock: *n* = 60; WCS417: *n* = 60), *myb15‐2* (Mock: *n* = 60; WCS417: *n* = 60), *pmr4‐1* (Mock: *n* = 60; WCS417: *n* = 60), *pad3‐1* (Mock: *n* = 60; WCS417: *n* = 60), *gtr1/2* (Mock: *n* = 60; WCS417: *n* = 55), *myb51* (Mock: *n* = 30; WCS417: *n* = 30), *myb34/51/122* (Mock: *n* = 60; WCS417: *n* = 60), *cyp79b2/b3* (Mock: *n* = 30; WCS417: *n* = 30), and *myb28/29* (Mock: *n* = 60; WCS417: *n* = 59). CFU, colony‐forming units.

To investigate the role of chemical and structural barriers in beneficial plant–microbe interactions, we utilized an *in vitro* system (Herrera Paredes *et al*., 2018) where Arabidopsis roots can be colonized by WCS417 mixed into the agar (Fig. S1a,b). Colonization of wild‐type (WT) Col‐0 roots by WCS417 promoted shoot growth (Fig. S1c,d) and changed root architecture by enhancing primary root length and lateral root formation (Fig. S1e,f). Next, we investigated the effect of WCS417 on shoot fresh weight, primary root length, and lateral root number of mutants affected in cutinization (*bdg‐1*, *dcr‐2*, and *gpat4/8*), suberization, and Casparian strip formation (*esb1*, *myb36‐2/sgn3‐3*, and *sgn3‐3*), lignification (*myb15‐1* and *myb15‐2*), callose deposition (*pmr4‐1*), camalexin (*pad3‐1*), and glucosinolate biosynthesis and transport (*myb28/29*, *myb51*, *myb34/51/122*, *cyp79b2/b3*, and *gtr1/2*; Fig. 1a; Table S4). The shoot fresh weight of *myb15‐2* and *pmr4‐1* showed no significant response to WCS417 treatment (Fig. S2a); the primary root length was either unaffected or even inhibited by WCS417 in all tested mutants except *sgn3‐3* (Fig. S2b); while the lateral root number of *myb15‐1*, *myb15‐2*, *pmr4‐1*, and *pad3‐1* was not significantly altered in response to WCS417 (Fig. S2c). To show different responses of Arabidopsis genotypes to WCS417 more clearly, we calculated the shoot fresh weight (Fig. 1b), primary root length (Fig. 1c), and lateral root number (Fig. 1d) relative to that of corresponding mock‐treated seedlings. Relative to Col‐0, which showed significant shoot and root growth promotion (1.58‐fold shoot weight, 1.41‐fold primary root length, and 1.81‐fold lateral root number) in response to WCS417, 11 mutants (*bdg‐1*, *dcr‐2*, *esb1*, *myb36‐2/sgn3‐3*, *myb15‐1*, *myb15‐2*, *pmr4‐1*, *pad3‐1*, *gtr1/2*, *myb51*, and *myb34/51/122*) displayed reduced WCS417‐mediated shoot growth promotion (fold change significantly < 1.58), while one suberization mutant (*sgn3‐3*) showed enhanced shoot growth promotion (fold change significantly > 1.58; Fig. 1b). All the tested mutants displayed reduced WCS417‐mediated primary root length promotion compared with Col‐0 (fold change significantly < 1.41; Fig. 1c). As for lateral root number, the beneficial influence of WCS417 relative to Col‐0 was significantly reduced in 5 of the 15 mutants (fold change significantly < 1.81; *bdg‐1*, *dcr‐2*, *myb15‐1*, *myb15‐2*, and *pad3‐1*) but enhanced in another suberization mutant *myb36‐2/sgn3‐3* and the callose deposition mutant *pmr4‐1* (fold change significantly > 1.81; Fig. 1d). These findings demonstrate that structural and chemical defense barriers can influence the beneficial effect of WCS417 on shoot growth and root architecture changes in Arabidopsis.

Notably, plant responses to WCS417 varied significantly across different genotypes. To further assess and compare the effects of treatment and genotype on Arabidopsis phenotypes, a PCA was conducted based on shoot weight, primary root length, and lateral root number as variables (Fig. S3). The PCA plot showed a separation between mock‐ and WCS417‐treated samples, indicating a consistent treatment effect across genotypes. To statistically validate these patterns, a PERMANOVA was performed, showing that treatment explained 6.1% (*R* ^2^ = 0.061, *P* = 0.001), genotype explained 21.1% of the total variation (*R* ^2^ = 0.211, *P* = 0.001), and their interaction contributed to a combined explained variance of 31.4% (*R* ^2^ = 0.314, *P* = 0.001). These results indicated that both factors could significantly affect Arabidopsis phenotypes, with genotype having a stronger effect.

### Root colonization levels and exudate‐supported growth rate of WCS417 across different genotypes

**Fig. 2:**
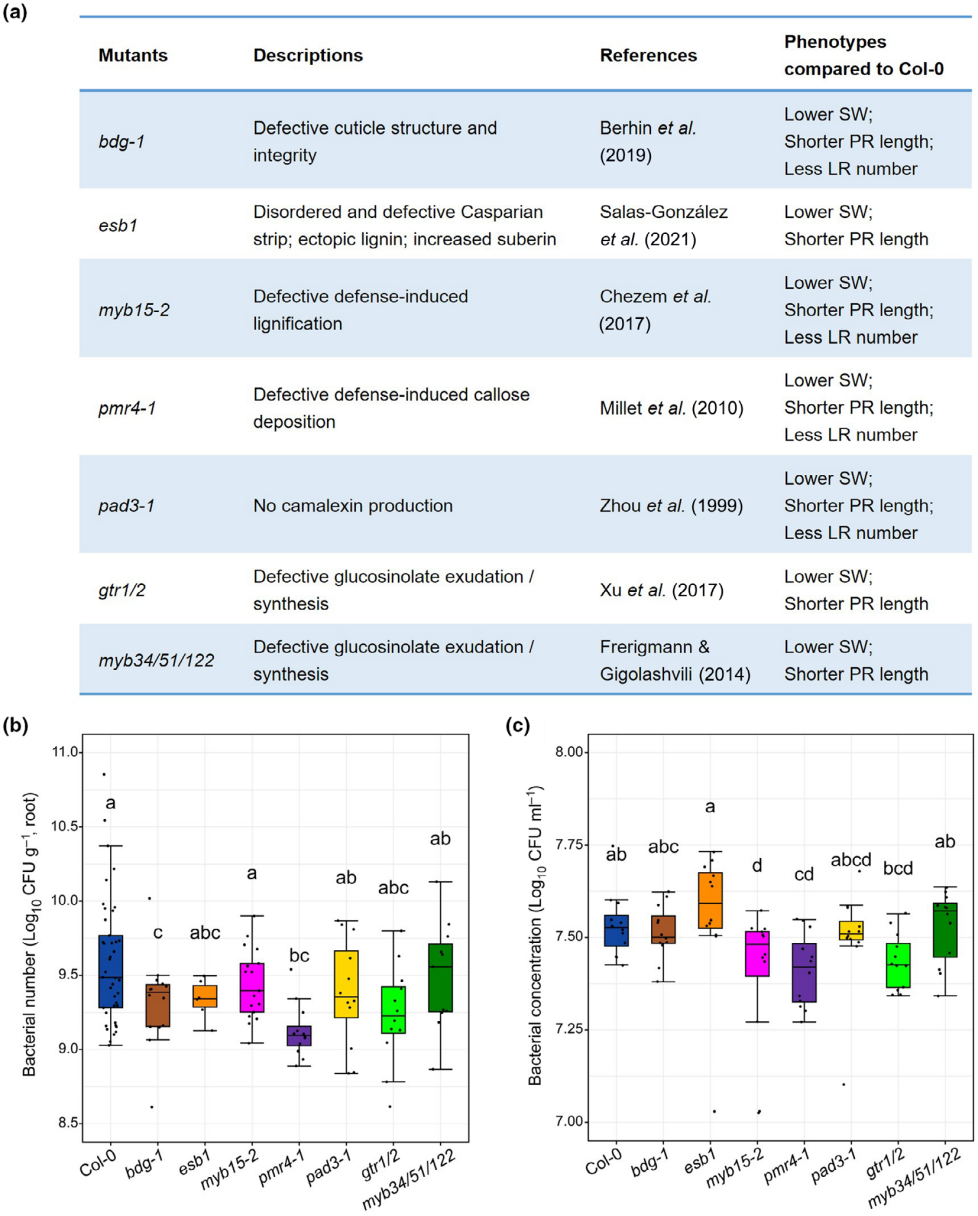
Selected defense barrier mutants display different effects on root colonization and growth of *Pseudomonas simiae* WCS417. (a) List of mutants selected according to their different growth phenotypes in response to WCS417 compared with *Arabidopsis thaliana* Columbia‐0 (Col‐0) (Fig. 1). (b) Colonization levels of WCS417 on roots of Arabidopsis Col‐0 and selected mutants at 7 d after seedlings were transplanted to Hoagland medium without sucrose containing 10^5^ CFU ml^−1^ WCS417. (c) Density of WCS417 cells after 24 h of growth in root exudates of the indicated Arabidopsis genotypes. In the boxplots, the horizontal line inside each box represents the median; the top and bottom edges of each box represent the 75^th^ and 25^th^ quartiles, respectively; and the upper and lower whiskers extend to 1.5× the interquartile range from the top and bottom of the box, respectively. Different lowercase letters represent significant differences among indicated genotypes (one‐way ANOVA with least significant difference test, *P* < 0.05). Each data point represents a biological replicate. In the case of bacterial root colonization, the number of biological replicates (*n*) per genotype was as follows: Col‐0 (*n* = 41), *bdg‐1* (*n* = 16), *esb1* (*n* = 6), *myb15‐2* (*n* = 18), *pmr4‐1* (*n* = 12), *pad3‐1* (*n* = 12), *gtr1/2* (*n* = 12), and *myb34/51/122* (*n* = 11). In the case of bacterial growth, 12 biological replicates (*n* = 12) were used for each genotype. LR, lateral root; PR, primary root; SW, shoot weight.

To further explore the effects of defense barriers on the WCS417‐Arabidopsis interaction, we focused on *bdg‐1* (cutin‐deficient mutant), *esb1* (endodermal barrier mutant), *myb15‐2* (lignification mutant), *pmr4‐1* (callose deposition mutant), *pad3‐1* (camalexin production mutant), *myb34/51/122* (glucosinolate synthesis deficient mutants) and *gtr1/2* (glucosinolate transport deficient mutant) for subsequent experiments (Fig. 2a). These mutants displayed more pronounced phenotypic alterations relative to Col‐0 than those observed in the other mutants following WCS417 inoculation and were representative of different defense barrier classes. First, WCS417 colonization levels were quantified on the roots of these mutants in an *in vitro* plate assay (Fig. S1a). We observed a significant reduction in the number of WCS417 cells on the roots of *bdg‐1* and *pmr4‐1* compared with Col‐0 (Fig. 2b), suggesting that reduced colonization of WCS417 could account for its reduced capacity to promote the growth of these mutants. However, other mechanisms might explain less growth promotion caused by WCS417 in *esb1*, *myb15‐2*, *pad3‐1*, *gtr1/2*, and *myb34/51/122*.

To examine how root exudates affect WCS417 growth, we collected root exudates from all seven genotypes and grew WCS417 in these exudates for 24 h. WCS417 grew significantly less in root exudates from *myb15‐2* and *pmr4‐1* compared with Col‐0 exudates (Fig. 2c), likely explaining the lower root colonization of *pmr4‐1* by WCS417. Interestingly, WCS417 colonization was not affected in *myb15‐2* (Fig. 2b), despite growing less in root exudates from this mutant (Fig. 2c). Moreover, WCS417 exhibited less colonization on *bdg‐1* roots compared with Col‐0, yet the exudates from this mutant did not affect its growth. These results indicate that root exudates from defense barrier mutants differentially influence WCS417 growth and colonization. Moreover, root exudates may target other bacterial colonization mechanisms as well.

### Root defense barriers affect transcriptional responses of WCS417

**Fig. 3:**
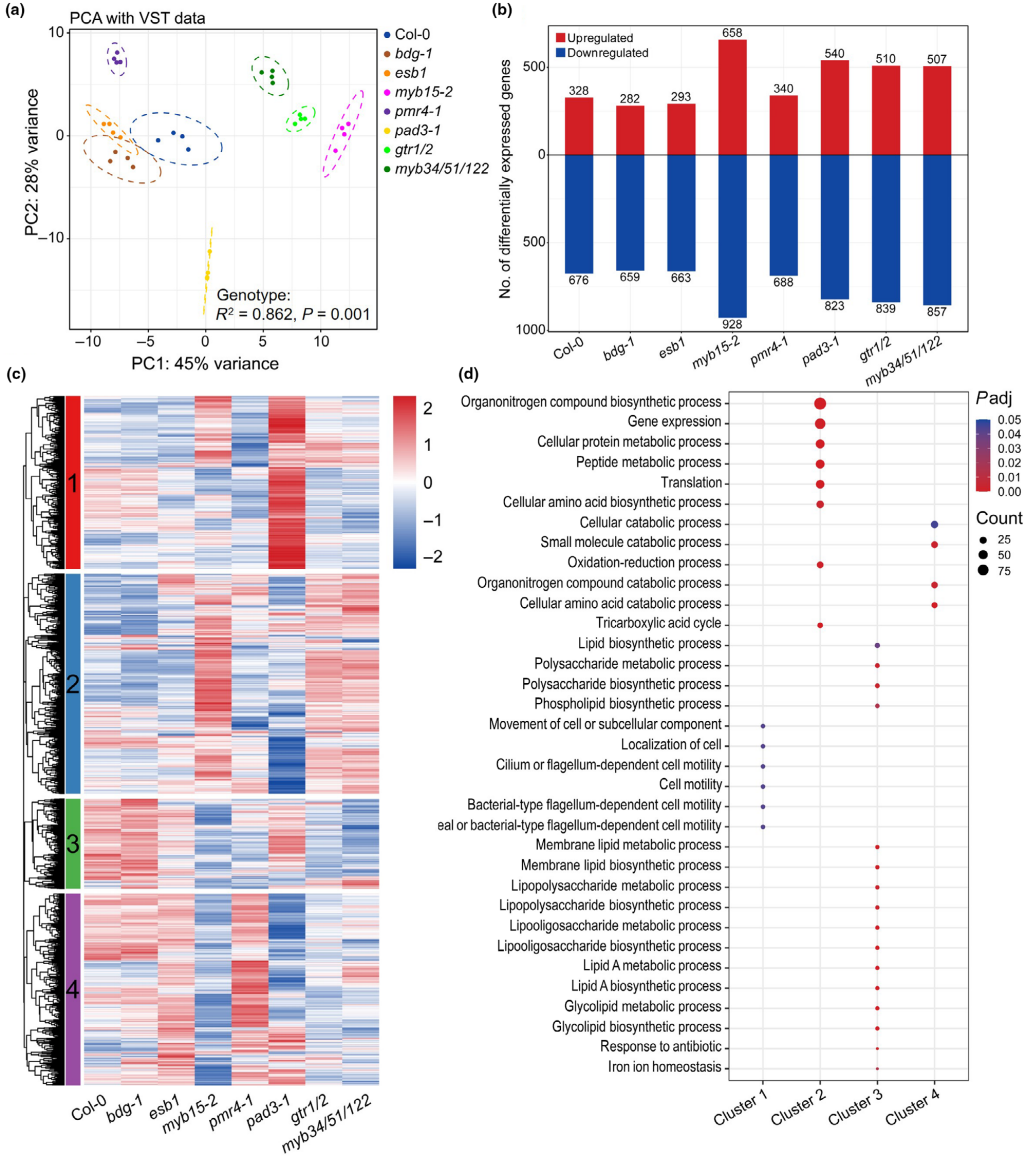
Effects of root exudates from defense barrier mutants on the transcriptome of *Pseudomonas simiae* WCS417. (a) Principal component analysis (PCA) of WCS417 transcriptional profiles in response to root exudates from *Arabidopsis thaliana* Columbia‐0 (Col‐0) and defense barrier mutants. Each color represents bacterial samples (*n* = 4) treated with root exudates from the indicated Arabidopsis genotypes. Ellipses correspond to *t*‐distributions fitted to each treatment (95% confidence interval). (b) Number of differentially expressed genes (DEGs) in WCS417 upon treatment with respective root exudates compared with mock (log_2_ fold change ≥ 1 or ≤ −1, false discovery rate (FDR) < 0.05). Red bars correspond to upregulated and blue bars to downregulated DEGs. (c) Expression pattern of 2196 DEGs in WCS417 in response to different root exudates. DEGs were divided into four clusters by *k*‐means. Each row of the heatmap represents an individual gene and indicates the level of expression, with red and blue indicating increased and decreased expression (standardized on a per gene basis across different root exudate treatments), respectively. (d) Bubble plot showing Gene Ontology (GO) terms enriched in each cluster. Dot color represents adjusted *P* values calculated using a hypergeometric test, with red indicating lower and blue indicating higher values. Dot size represents the count of genes enriched in each GO term, with larger ones indicating more genes and smaller ones indicating fewer genes.

To investigate the effect of root exudates on bacterial activity, WCS417 was grown in root exudates from Col‐0 and the seven selected root barrier mutants for 1 h, after which we profiled its transcriptome by RNA sequencing. The PERMANOVA of the WCS417 RNA sequencing data (Fig. S4a) shows a clear separation between the mock (Milli‐Q water) and the root exudate treatments (*R* ^2^ = 0.926, *P* = 0.001), indicating a clear transcriptional response of WCS417 to Arabidopsis root exudates. Gene Ontology (GO) enrichment analysis of the top 1000 genes driving the sample separation in the PC1 component, which explains 68% of total variation (Fig. S4a), identified enriched processes related to translation, organelle formation, and bacterial movement (Fig. S4b). The fact that several genes are associated with bacterial motility could indicate that root exudates influence WCS417 motility. Additionally, the transcriptional profiles of WCS417 varied significantly between exudates from Col‐0 and the mutants (Fig. 3a; *R* ^2^ = 0.862, *P* = 0.001).

We identified a total of 2196 DEGs (log_2_ fold change ≥ 1 or ≤ −1 and an FDR < 0.05) in WCS417 exposed to root exudates compared with mock (Fig. 3b; Table S5). The number of upregulated DEGs ranged between 282 and 658, while the number of downregulated DEGs ranged between 659 and 928, with root exudates of *myb15‐2* causing the highest number of DEGs in WCS417. *K*‐means clustering of the 2196 DEGs revealed four clusters with distinct gene expression patterns (Fig. 3c). The expression pattern of Cluster 1 (563 DEGs) displayed a strong upregulation by exudates from camalexin biosynthesis mutant *pad3‐1* compared with those from Col‐0 and other mutants. Subsequent GO enrichment analysis (Fig. 3d; Table S6) revealed that the genes in Cluster 1 were predominantly associated with bacterial motility, suggesting camalexin as a potential motility inhibitor of WCS417. The genes in Cluster 2 (716 DEGs), which showed higher expression to root exudates from *myb15‐2*, *gtr1/2*, and *myb34/51/122*, were mainly related to nitrogen metabolism. Cluster 3 (293 DEGs) exhibited significantly reduced expression of WCS417 genes after exposure to root exudates from *myb15‐2*, *pmr4‐1*, *gtr1/2*, and *myb34/51/122*, compared with Col‐0 root exudates. These genes were primarily linked to lipid and polysaccharide metabolism. Finally, Cluster 4 genes (624 DEGs), which were most strongly downregulated in WCS417 by root exudates from *myb15‐2*, *pad3‐1*, *gtr1/2*, and *myb34/51/122* compared with Col‐0, were primarily associated with nitrogen and small molecule catabolism. Overall, these results demonstrate that root exudates from structural and chemical defense barrier mutants significantly influence the transcriptional activity of WCS417, and potentially impact its colonization and functionality in the rhizosphere.

### Camalexin inhibits motility and chemotaxis of WCS417

**Fig. 4:**
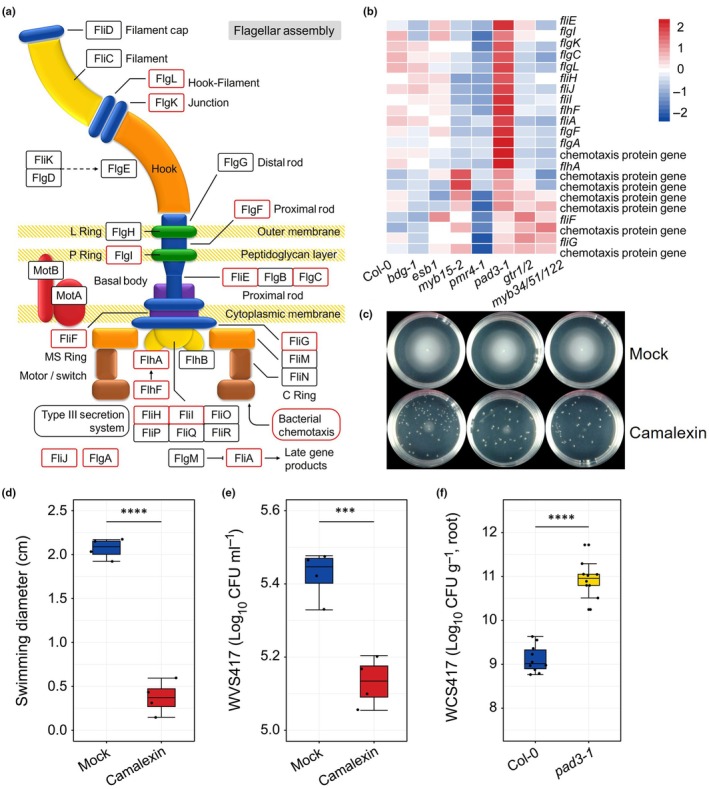
Impact of camalexin on bacterial motility‐related gene expression, and on swimming and chemotaxis of *Pseudomonas simiae* WCS417. (a) The flagellar assembly of WCS417 modified from Fitness Browser (https://fit.genomics.lbl.gov/cgi‐bin/keggmap.cgi?mapId = 02040&orgId = WCS417). Genes in red boxes are in Cluster 1 in Fig. 3(c). (b) Expression patterns of genes encoding building blocks of the bacterial flagellum and proteins involved in chemotaxis in WCS417 in response to different root exudates. Each row of the heatmap represents an individual gene and indicates the level of expression, with red and blue indicating increased and decreased expression levels, respectively (standardized on a per gene basis across different root exudates). (c) Size of WCS417 colonies after 20 h of growth on semi‐solidified medium supplemented with dimethyl sulfoxide (DMSO) (Mock) or 1 mM camalexin. (d) Swimming diameter of WCS417 after 20 h of growth on semi‐solidified medium supplemented with DMSO (Mock) or 1 mM camalexin. Asterisks represent statistically significant differences between treatments (Student's *t*‐test; ****, *P* < 0.0001, *n* = 4). (e) The log_10_‐transformed number of WCS417 cells attracted by DMSO (Mock) or 1 mM camalexin. Asterisks represent statistically significant differences between treatments (Student's *t*‐test; ***, *P* < 0.001, *n* = 4). (f) Colonization levels of WCS417 on roots of 25‐d‐old *Arabidopsis thaliana* Columbia‐0 (Col‐0) and camalexin biosynthesis mutant *pad3‐1* grown in soil. In the boxplots, the horizontal line inside each box represents the median; the top and bottom edges of each box represent the 75^th^ and 25^th^ quartiles, respectively, and the upper and lower whiskers extend to 1.5× the interquartile range from the top and bottom of the box, respectively. Asterisks represent statistically significant differences between Col‐0 and *pad3‐1* (Student's *t*‐test; ****, *P* < 0.0001, *n* = 10). Each data point represents a biological replicate.

To validate one of the most prominent effects of root exudates on WCS417 gene expression, we focused on the expression profile of Cluster 1 DEGs in response to exudates of camalexin mutant *pad3‐1* (Fig. 3c). Cluster 1 contains 15 genes encoding building blocks of the bacterial flagellum and seven genes encoding proteins involved in chemotaxis (Fig. 4a). More importantly, all these genes in WCS417 were upregulated in response to the root exudates of *pad3‐1* compared with Col‐0 (Fig. 4b). These results indicate that camalexin, among other metabolites, in root exudates might suppress genes involved in bacterial motility and chemotaxis.

To investigate this, we tested the effect of camalexin on the swimming ability of WCS417 on semi‐solidified agar plates. Camalexin significantly inhibited the swimming diameter of WCS417 (Fig. 4c,d), confirming its inhibitory effect on bacterial motility. We further assessed camalexin's effect on the chemotactic response of WCS417 using a quantitative capillary method (Fig. S5). The number of WCS417 bacteria entering the capillary was significantly inhibited by camalexin (Fig. 4e), indicating that WCS417 is repelled by camalexin. Although WCS417 colonization of *pad3‐1* roots was similar to Col‐0 *in vitro* (Fig. 2b), *pad3‐1* plants showed significantly higher WCS417 colonization in soil‐grown conditions (Fig. 4f), highlighting a strong effect of the *pad3* mutation on bacterial colonization specifically in the soil environment. More interestingly, camalexin also significantly inhibited the swimming of *Pst* DC3000, *Rhizobium* sp. YAF28, and *B. subtilis* GB03 (Fig. S6), indicating a broader inhibitory effect on the motility of different bacterial genera. Together, these findings align with the bacterial gene expression profiles demonstrating that camalexin in root exudates negatively affects bacterial motility and chemotaxis.

It is important to note that in the motility assay (Fig. 4c), camalexin was uniformly mixed into the agar‐solidified medium. This experimental design indicates that the reduced motility observed is primarily due to direct inhibition of bacterial swimming, rather than a repellent effect. By contrast, the capillary assay (Fig. S5) established a camalexin gradient, resulting in a clear avoidance response (Fig. 4e). Consistent with these observations, genes associated with both motility and chemotaxis in WCS417 were upregulated in response to root exudates from the *pad3‐1* mutant, which is deficient in camalexin biosynthesis (Fig. 4b). Together, these findings suggest that camalexin both suppresses motility and acts as a chemorepellent under different conditions.

### Distinct root exudate profiles in Arabidopsis Col‐0 and defense barrier mutants

Our results demonstrated that root exudates from different Arabidopsis genotypes differentially influence the transcriptomic responses of WCS417 (Fig. 3). To identify candidate components in exudates driving these bacterial responses, untargeted metabolomic profiling was performed. PLS‐DA of log‐transformed feature intensities revealed clear separation among all root exudate samples (Fig. S7a), providing a basis for understanding the divergent transcriptional patterns observed in WCS417 (Fig. 3a). Compared with Col‐0, the number of features with significantly altered abundance in the exudates of *bdg‐1*, *esb1*, *myb15‐2*, *pmr4‐1*, *pad3‐1*, *gtr1/2*, and *myb34/51/122* was 317, 189, 93, 475, 116, 82, and 97, respectively (Fig. S7b). Notably, *pmr4–1* exhibited the greatest number of differential features, followed by *bdg‐1*. Both mutants are impaired in structural barriers, likely resulting in enhanced passive metabolite leakage into the rhizosphere.

Annotation of these differential features yielded 86 putative metabolites across all the genotypes (Fig. S7c). Most annotated features were enriched in *pmr4–1* exudates compared with Col‐0 and other mutants, supporting that the defective callose deposition promotes more metabolite exudation. The distinct exudate profile of *pmr4–1* aligns with its distinctive induction of WCS417 gene expression (Fig. 3a). Conversely, the root exudates of *bdg‐1* and *esb1* were generally depleted in these features relative to Col‐0, consistent with the similar transcriptomic responses they induced in WCS417 (Fig. 3a).

### Differential root transcriptome changes in Arabidopsis defense barrier mutants upon WCS417 colonization

**Fig. 5:**
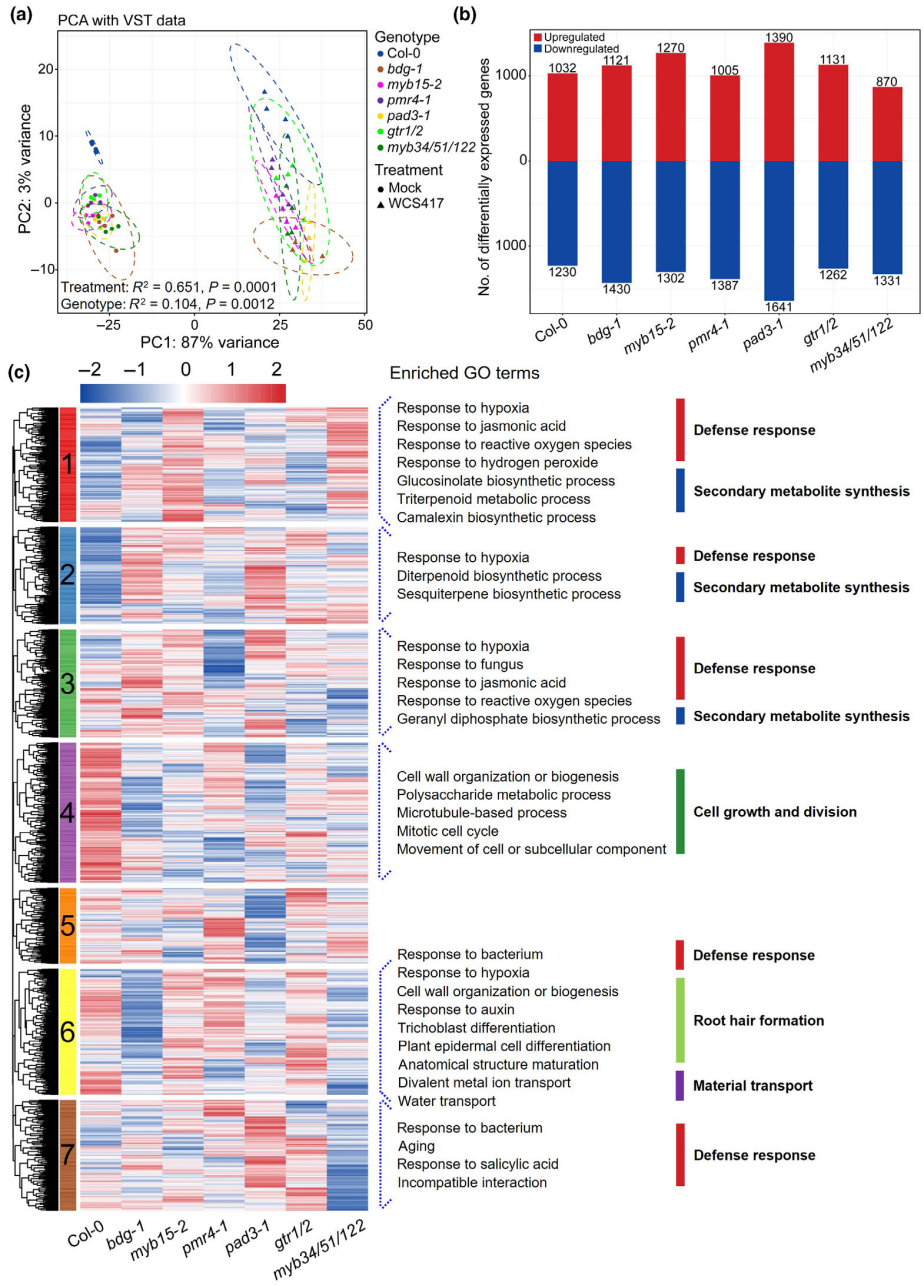
Root transcriptional responses of *Arabidopsis thaliana* Columbia‐0 (Col‐0) and root defense barrier mutants to *Pseudomonas simiae* WCS417 colonization. (a) Principal component analysis plot of gene expression profiles of roots of indicated Arabidopsis genotypes, 6 h after treatment with either 10 mM MgSO_4_ (Mock) or WCS417. Different colors represent different genotypes and different shapes represent Mock or WCS417 treated samples (*n* = 4). Ellipses correspond to *t*‐distributions fitted to each genotype (95% confidence interval). (b) Number of differentially expressed genes (DEGs) in different genotypes upon the treatment with WCS417 compared with MgSO_4_. Red bars correspond to upregulated (log_2_ fold change ≥ 1.5, false discovery rate (FDR) < 0.01) and blue bars to downregulated (log_2_ fold change ≤ −1.5, FDR < 0.01) DEGs generated by the DESeq2 package in R. (c) Expression patterns of 4332 DEGs in roots of different genotypes in response to WCS417 (log_2_ fold change ≥ 1.5 or ≤ −1.5, FDR < 0.01) and Gene Ontology (GO) term enrichment analysis of all the DEGs. DEGs are divided into seven clusters by *k*‐means. Each row of the heatmap represents an individual gene and indicates levels of expression, with red and blue indicating increased and decreased expression (standardized on a per gene basis across different genotypes), respectively. Enriched GO terms in Clusters 1–4 and 6–7 are listed and grouped into five categories: defense response, (defense‐related) secondary metabolite synthesis, cell growth and division, root hair formation and material transport. Different categories are color‐coded for clarity. GO terms with adjusted *P* values < 0.05 are shown as the enriched ones and adjusted *P* values are calculated using a hypergeometric test.

To investigate how the selected defense barriers impact root responses to WCS417 colonization, we profiled the early transcriptional changes in Col‐0 and seven barrier mutants. Roots of 17‐d‐old seedlings were treated with WCS417 or with 10 mM MgSO_4_ (mock) and root gene expression was profiled 6 h postinoculation using RNA sequencing. The impact of WCS417 on root transcriptomes across the different Arabidopsis genotypes was visualized using PCA, where PC1 explained 87% of the total variation and clearly separated the mock‐ and WCS417‐treated samples (Fig. 5a). PERMANOVA analysis demonstrated the significant influence of WCS417 on root transcriptomes (*R* ^2^ = 0.651, *P* = 0.0001) and among the Arabidopsis genotypes (*R* ^2^ = 0.104, *P* = 0.0012). To further assess genotype‐specific effects, mock‐ and WCS417‐treated samples were analyzed separately. In a PCA analysis of mock‐treated samples (Fig. S8a), Col‐0 clearly separated from the defense mutants, which clustered more closely together. Conversely, WCS417‐treated samples showed greater divergence among genotypes (Fig. S8b), consistent with the PERMANOVA results, where the *R* ^2^ values increased from 0.365 to 0.477 following WCS417 treatment. These results demonstrate that Col‐0 and the seven defense barrier mutants exhibit differential responses to WCS417 colonization, highlighting the importance of each defense barrier in this plant–microbe interaction.

Next, we identified 4332 DEGs (log_2_ fold change ≥ 1.5 or ≤ −1.5 and an FDR < 0.01) between mock‐ and WCS417‐treated roots of each genotype (Table S7). Across genotypes, 870–1390 DEGs were upregulated and 1230 to 1641 were downregulated. Notably, the camalexin biosynthesis mutant *pad3‐1* displayed the most DEGs in response to WCS417 colonization (Fig. 5b). Additionally, an UpSet plot showed that there were 1177 DEGs common to all the genotypes, along with 155, 142, 109, 176, 277, 102, and 71 DEGs unique to Col‐0, *bdg‐1*, *myb15‐2*, *pmr4‐1*, *pad3‐1*, *gtr1/2*, and *myb34/51/122*, respectively (Fig. S9). *K*‐means clustering of all DEGs from different genotypes resulted in seven gene clusters (Fig. 5c). Clusters 2 and 4 showed distinct expression patterns: Cluster 2 genes were significantly less expressed while Cluster 4 genes were more expressed in WCS417‐treated Col‐0 compared with the mutants (Figs 5c, S10). GO term analysis revealed that Cluster 2 genes were primarily involved in defense responses and secondary metabolite synthesis, while Cluster 4 genes were related to cell growth and division (Fig. 5c; Table S8). The weaker or opposite expression patterns of Clusters 2 and 4 genes in most root defense mutants compared with Col‐0 suggest that disrupting specific root defense components alters the balance between plant growth and defense during WCS417 colonization.

At a more detailed level, Cluster 1 and Cluster 3 DEGs behaved clearly differently from Col‐0 in all the tested mutants; Cluster 5 DEGs in *bdg‐1*, *myb15‐2*, *pmr4‐1*, *pad3‐1*, and *gtr1/2*; Cluster 6 DEGs in *bdg‐1*, *pmr4‐1*, *pad3‐1*, *gtr1/2*, and *myb34/51/122*; and Cluster 7 DEGs in *myb15‐2*, *pmr4‐1*, *pad3‐1*, *gtr1/2*, and *myb34/51/122* (Figs 5c, S10). GO enrichment analysis for these clusters also showed an overrepresentation of genes associated with defense responses (including responses to hypoxia, ROS, jasmonic acid, and salicylic acid as well as responses to fungi and bacteria), (defense‐related) secondary metabolite synthesis, root hair formation, and material transport. This supports the notion that the balance between certain plant growth and defense responses is shifted in the root defense barrier mutants, which could explain why the beneficial effects of WCS417 on plant growth and root architecture changes are less pronounced in these mutants compared with Col‐0 (Fig. 1).

### Differential ROS production in Arabidopsis defense barrier mutants upon WCS417 colonization

**Fig. 6:**
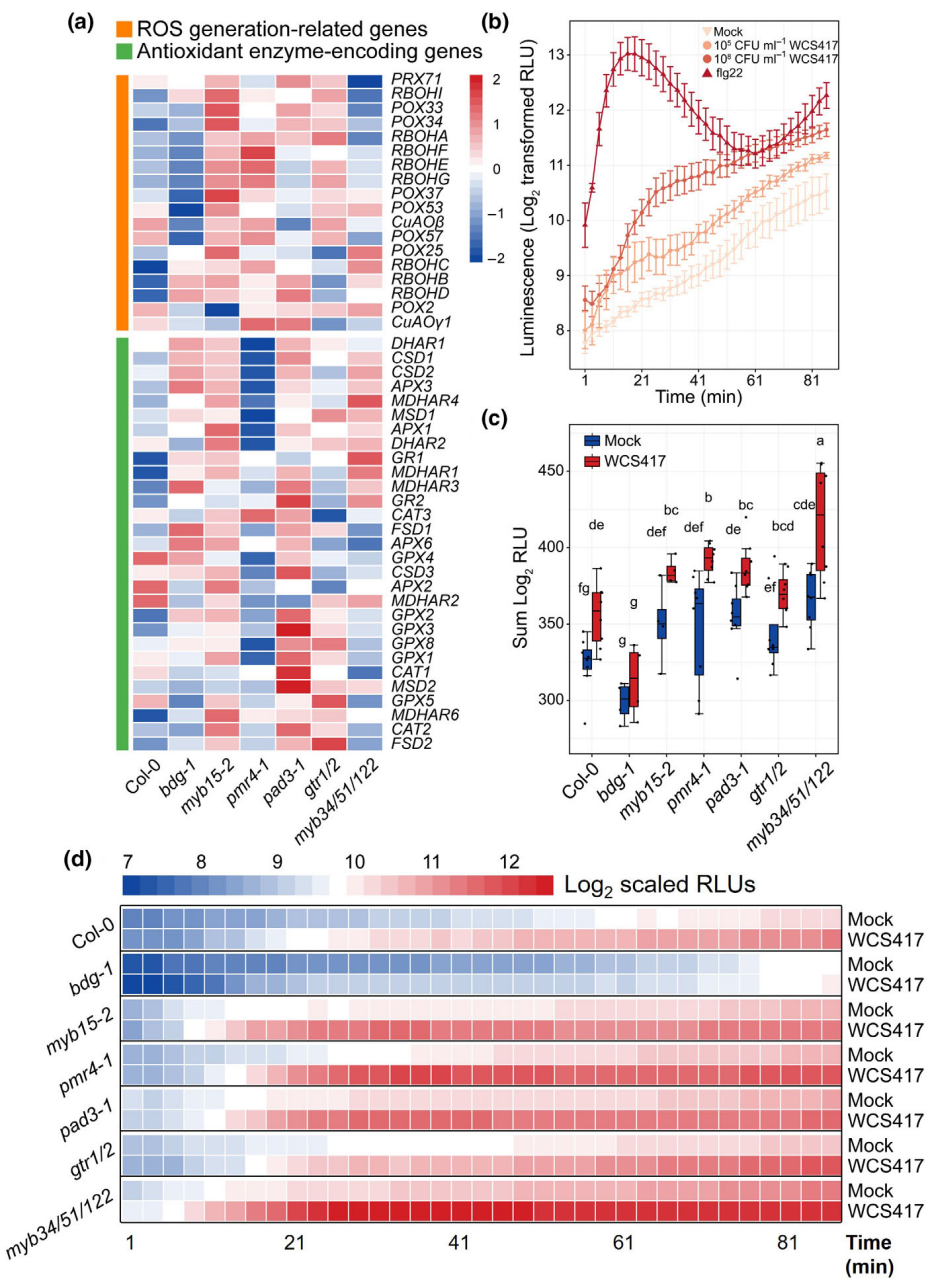
Reactive oxygen species (ROS) production triggered by *Pseudomonas simiae* WCS417 in the roots of *Arabidopsis thaliana* Columbia‐0 (Col‐0) and selected defense component mutants. (a) Expression patterns of representative ROS generation‐related and antioxidant enzyme‐encoding genes in roots of indicated Arabidopsis genotypes in response to colonization of the roots by WCS417. Each row of the heatmap represents an individual gene and indicates the level of expression, with red and blue indicating increased and decreased expression levels, respectively (standardized on a per gene basis across different genotypes). (b) ROS burst in the roots of Arabidopsis Col‐0 in response to 10 mM MgSO4 (Mock), 10^5^ or 10^8^ CFU ml^−1^ WCS417, or 0.2 μM flg22 treatment. Values are mean and SE of log_2_ scaled relative luminescence units (RLUs; Mock: *n* = 8; 10^5^ CFU ml^−1^ WCS417: *n* = 4; 10^8^ CFU ml^−1^ WCS417: *n* = 8; 0.2 μM flg22: *n* = 4). ROS generation in roots is indicated by the luminescence induced by L‐012, which was recorded on a luminometer at 2.5 min intervals and for 86 min. The error bars represent standard error (SE). (c) Total ROS produced in roots of indicated Arabidopsis genotypes after 86 min of mock or WCS417 treatment. Values are sums of log_2_ scaled RLU after treatment of the roots with either 10 mM MgSO4 (Mock) or 10^8^ CFU ml^−1^ WCS417. Statistical analysis was performed using two‐way ANOVA to assess the individual and collaborative effects of genotype and treatment. *Post hoc* comparisons were conducted using the least significant difference (LSD) test. In the boxplots, the horizontal line inside each box represents the median; the top and bottom edges of each box represent the 75^th^ and 25^th^ quartiles, respectively; and the upper and lower whiskers extend to 1.5× the interquartile range from the top and bottom of the box, respectively. Different lowercase letters indicate statistically significant differences among different genotypes and treatments (*P* < 0.05). Each data point represents a biological replicate and the number of biological replicates (*n*) per genotype and treatment was as follows: Col‐0 (Mock: *n* = 8; WCS417: *n* = 8), *bdg‐1* (Mock: *n* = 4; WCS417: *n* = 4), *myb15‐2* (Mock: *n* = 4; WCS417: *n* = 4), *pmr4‐1* (Mock: *n* = 8; WCS417: *n* = 8), *pad3‐1* (Mock: *n* = 8; WCS417: *n* = 8), *gtr1/2* (Mock: *n* = 8; WCS417: *n* = 8), and *myb34/51/122* (Mock: *n* = 8; WCS417: *n* = 8). (d) Heatmap showing ROS generation in roots of indicated Arabidopsis genotypes in response to 10 mM MgSO_4_ (Mock) or WCS417 treatment. Values are mean of log_2_ scaled RLUs after treatment of the roots with either 10 mM MgSO_4_ (Mock) or 10^8^ CFU ml^−1^ WCS417 (*n* = 4 or 8). Low levels of ROS production are shown in blue and high levels are shown in red.

Among the enriched GO terms associated with DEGs that differed in WCS417‐treated defense barrier mutants, ‘Response to hypoxia’, ‘Response to reactive oxygen species (ROS)’, or ‘Response to hydrogen peroxide’ appeared in 4 of the 7 DEG clusters (Fig. 5c). This suggests a shift in the role of ROS, typically a marker for plant defense activation (Mittler *et al*., 2022), in the root responses to WCS417 in these mutants compared with Col‐0. To further investigate this, we assessed the expression of 18 ROS generation‐related and 29 antioxidant enzyme‐encoding genes (Mittler *et al*., 2004; Podgórska *et al*., 2017) in roots of the different Arabidopsis genotypes following WCS417 root colonization (Fig. 6a). In response to WCS417 most ROS generation‐related genes were expressed at lower levels in Col‐0, but generally higher in *myb15‐2*, *pmr4‐1*, *pad3‐1*, and *gtr1/2*. Similarly, major antioxidant enzyme‐encoding genes showed lower expression in Col‐0, but higher levels in *bdg‐1*, *myb15‐2*, *pad3‐1*, and *gtr1/2*. Specifically, the expression levels of *RBOHB* and *RBOHD*, which play essential roles in plant immunity (Wang *et al*., 2020), were relatively higher in *bdg‐1*, *myb15‐2*, *pmr4‐1*, *pad3‐1*, and *myb34/51/122* than in Col‐0. This suggests that these root defense barrier mutants may exhibit a stronger ROS burst in response to root colonization by WCS417 than Col‐0.

To verify this experimentally, we measured ROS production in mock‐ and WCS417‐inoculated roots of all genotypes, using L‐012 as a luminol‐based chemiluminescent probe to superoxide. As a positive control, we used the bacterial MAMP flg22, which induced a fast and strong ROS burst in WT Col‐0 roots (Fig. 6b). WCS417 bacteria also induced ROS production in Col‐0 roots, which was dose‐dependent but overall lower than that induced by flg22 (Fig. 6b). We then tested ROS production in the roots of the seven defense barrier mutants in response to the highest dose of WCS417 (10^8^ CFU ml^−1^). We determined the total ROS accumulation in the roots of each genotype by calculating the sum of log_2_ relative luminescence units (RLUs; Fig. 6c). The results show that *myb15‐2*, *pmr4‐1*, *pad3‐1*, and *myb34/51/122* accumulated statistically more ROS in their roots in response to WCS417 compared with Col‐0. Additionally, compared with Col‐0, WCS417 induced a faster and stronger ROS burst in all the tested mutants except *bdg‐1*, which displayed a clearly lower production of ROS in response to WCS417 (Fig. 6d). These observations are consistent with the above‐described ROS‐related gene expression profiles and suggest that most of the selected defense barrier mutants develop a faster and stronger ROS burst in their roots in response to colonization by WCS417. Considering the role of ROS in mediating the activation of plant defense responses at both local and systemic tissues, in most cases not favoring growth (Torres *et al*., 2006; Huot *et al*., 2014), this stronger ROS response may contribute to the shifted balance between growth and defense responses observed in the roots of the WCS417‐treated mutants.

## Discussion

Plant defense barriers can locally influence the interaction between plants and pathogens in the roots (De Coninck *et al*., 2015; Pascale *et al*., 2020; Fröschel *et al*., 2021; Verbon *et al*., 2023). However, their role in the interplay between plants and beneficial microbes is largely uncharacterized. In this study, we used WCS417, a model beneficial rhizobacterium (Pieterse *et al*., 2021), to investigate how different root defense barriers (Fig. 1a) affect its ability to induce beneficial phenotypes on Arabidopsis (Fig. 1b–d). Our results showed that when structural barriers (such as cutin, suberin, callose, and lignin) or chemical defenses (including camalexin and glucosinolate) are impaired, the WCS417‐mediated shoot and/or root growth responses are attenuated (Fig. 1). This reduction in beneficial effects could be related to decreased bacterial colonization on the roots of defense barrier mutants (Fig. 2b) or alterations in bacterial functions in response to root exudates of these mutants (Fig. 3d), but also to disrupted signaling and changes in bacterial perception. These results suggest that cutinization, suberization, callose deposition, lignification, and the synthesis/exudation of camalexin and glucosinolates in Arabidopsis roots play supportive roles in facilitating beneficial interactions with WCS417.

### Effect of dysfunctional defense barriers on growth and defense

When defense barriers are compromised in the root, the beneficial effects of WCS417 on Arabidopsis shoot growth and root architecture changes are attenuated (Fig. 1). Although beneficial microbes are known to promote plant growth and development (Finkel *et al*., 2017; Pieterse *et al*., 2021), plants must also regulate the overgrowth and potential detrimental effects of their microbial partners by activating proper defense mechanisms (Pascale *et al*., 2020). For example, the ROS burst, a typical plant defense response (Mittler *et al*., 2022), can limit the colonization of beneficial *Pseudomonas* species in the Arabidopsis rhizosphere (Song *et al*., 2021). Additionally, the suberin‐coated endodermal barrier physically prevents the mutualistic endophyte *Serendipita indica* from entering the vascular stele of Arabidopsis (Fröschel *et al*., 2021) and significantly impacts the colonization of roots by beneficial WCS417 bacteria (Verbon *et al*., 2023). Therefore, the activation of proper defense responses is indispensable to maintain the beneficial interactions between Arabidopsis and WCS417.

When plants allocate more resources to activate defense responses, plant growth can be stunted. For example, when Arabidopsis roots perceive MAMPs, genes related to plant immunity are upregulated while genes related to plant growth and development are downregulated (Stringlis *et al*., 2018a). In Col‐0, WCS417 primarily upregulated cell growth and division‐related genes (Fig. 5c). However, in defense barrier mutants, WCS417 predominantly upregulated genes associated with defense responses and (defense‐related) secondary metabolite synthesis (Fig. 5c). This aligns with the attenuated shoot growth and root development promotion observed in these mutants compared with Col‐0 (Fig. 1), as well as the faster and stronger ROS burst in the roots of several mutants in response to WCS417, despite inherent differences in ROS levels across genotypes (Fig. 6c). We speculate that the enhanced activation of defense responses in certain defense barrier mutants upon WCS417 colonization, compared with Col‐0 (Figs 5c, 6), might serve to compensate for the loss of the corresponding defense barriers. However, this compensatory mechanism likely comes at the expense of growth‐related processes in these mutants.

### Effect of antimicrobial secondary metabolites on the plant‐WCS417 interaction

Here, we observed that genes related to the synthesis of glucosinolates, terpenoids, and camalexin were upregulated in both structural and chemical defense barrier mutants compared with Col‐0 (Fig. 5c). Glucosinolates (Bressan *et al*., 2009), terpenoids (Huang *et al*., 2019), and camalexin (Koprivova *et al*., 2019) are known to influence plant–microbe interactions.

Camalexin, produced by Arabidopsis and other Brassicaceae plants, plays an important role in chemical defenses against pathogens (Ryffel *et al*., 2016). Previous studies have shown the antimicrobial activity of camalexin against *Pst* DC3000 (Kempthorne *et al*., 2021) and fungal pathogens (Sellam *et al*., 2007). We observed that camalexin inhibits the swimming motility of WCS417 (Fig. 4c,d), similar to the effects of coumarins on bacterial behavior (Yu *et al*., 2021; Table S9). Notably, camalexin showed a general inhibitory effect on the swimming of several other bacterial strains, including *Pst* DC3000 and representative rhizosphere colonizers (Fig. S6). In soil‐grown Arabidopsis seedlings, we recovered a greater number of WCS417 CFUs from the roots of camalexin biosynthesis mutant *pad3‐1* compared with Col‐0 (Fig. 4f). Interestingly, we did not observe more WCS417 CFUs in the roots of *pad3‐1* in the *in vitro* experimental system (Fig. 2b). There might be two explanations for this: (1) it is possible that under axenic *in vitro* conditions, reduced camalexin production reflected the influence of the microbiota on plant defense and metabolism, similar to their capacity to modulate the deposition of root diffusion barriers in the endodermis (Salas‐González *et al*., 2021), and (2) the age of plants is different in the soil (25 d old) and the *in vitro* (14 d old) system, and studies have shown a developmental effect on exudation (Zhalnina *et al*., 2018). Considering that a previous study showed camalexin is required for growth promotion in Arabidopsis during its interaction with another plant growth‐promotion bacterium, *Pseudomonas* sp. CH267 (Koprivova *et al*., 2019), it is possible that camalexin can modulate the beneficial Arabidopsis‐WCS417 interaction by preventing excessive bacterial colonization on the roots.

Besides camalexin, Brassicaceae plants produce another class of chemically diverse nitrogen‐ and sulfur‐containing secondary metabolites known as glucosinolates (Nambiar *et al*., 2021), which have been reported to influence the structure and composition of the Arabidopsis root‐associated microbiome (Bressan *et al*., 2009). MYB34, MYB51, and MYB122 are key transcription factors involved in the biosynthesis of indolic glucosinolates (Frerigmann & Gigolashvili, 2014). We observed that one indolic glucosinolate, 4‐methoxyglucobrassicin, was significantly depleted in the root exudate of *myb34/51/122* (Fig. S7c). Arabidopsis mutants lacking these three transcription factors exhibit increased susceptibility to the fungal pathogen *Plectosphaerella cucumerina* (Frerigmann *et al*., 2016). Consequently, the reduced beneficial effects of WCS417 observed in the *myb34/51/122* mutant may stem from the inability of this mutant to properly synthesize glucosinolates and then regulate its response to the bacterium. Moreover, GTR1 and GTR2 are glucosinolate‐specific transporters (Nour‐Eldin *et al*., 2012). Knockdown of GTR2 enhanced the plant defense responses of *Brassica juncea* (Nambiar *et al*., 2021), which was in line with the observation in this study (Figs 5c, 6). The enhanced defense responses may at the expense of growth‐related processes in *gtr1/2* (Fig. 1).

In general, both structural and chemical defense components play crucial roles in regulating secondary metabolism in Arabidopsis roots and then their exudates (Fig. S7), albeit through different mechanisms. These components, in turn, influence the growth, colonization, and functions of WCS417 and then its influences on Arabidopsis. Considering that the defects in defense barriers also impact plant growth and root architectures (Fig. 1), future studies should explore how differences in plant phenotype could affect both the composition and quantities of exuded metabolites.

### Structural defense components affecting root exudates and microbial interactions

We observed reduced colonization of WCS417 on the roots of *bdg‐1* and *pmr4‐1* compared with Col‐0 (Fig. 2b). Additionally, WCS417 grew less in root exudates of *myb15‐2* and *pmr4‐1* (Fig. 2c). The deficiencies in structural defense components might lead to increased leakage of nutrients as well as antimicrobial metabolites into the rhizosphere or changes in the plant ionome (Salas‐González *et al*., 2021), potentially affecting the beneficial interplay between Arabidopsis and WCS417. Metabolomic analysis revealed that root exudates of several structural defense barrier mutants contained significantly depleted or enriched levels of sugars (e.g. fructose and _D_‐maltose), organic acids (e.g. _L_‐malic acid), and amino acids (e.g. _D_‐alanine) compared with those of Col‐0 (Fig. S7c). These findings suggest that variations in the nutrient profile of root exudates and in their carbon content may influence the growth and functions of WCS417, thereby shaping its interaction with Arabidopsis.

During infection, the *pmr4‐1* mutant is unable to induce callose deposition at cell wall appositions (Nishimura *et al*., 2003). Consequently, most differential features were detected in the root exudates of *pmr4‐1* compared with Col‐0 (Fig. S7b). Notably, several annotated features with antimicrobial activity were enriched in its root exudates, such as benzoic acid, anthranilic acid, and *p*‐coumaraldehyde (Fig. S7c). These findings may explain the reduced growth of WCS417 in the root exudates of *pmr4‐1* compared with Col‐0, as well as its decreased colonization on *pmr4‐1* roots (Fig. 2). Moreover, it could help to understand why *pmr4‐1* exhibited increased resistance to fungal pathogens (Nishimura *et al*., 2003).

Cutin is primarily deposited on the outermost surface of the cell wall (Fich *et al*., 2016). The cutin‐deficient mutant *bdg‐1* exhibits increased Chl leakage from intact leaves compared with Col‐0 (Kurdyukov *et al*., 2006), indicating that altered barrier properties may affect the release of metabolites from roots and potentially affect bacterial colonization (Fig. 2b). Similarly, this cutin‐defective mutant displayed enhanced resistance to *Botrytis cinerea* in Arabidopsis (Tang *et al*., 2007). Moreover, the increased accumulation of cutin monomers may act as signaling molecules (Kurdyukov *et al*., 2006), which may lead to the upregulation of terpenoid synthesis‐related genes in *bdg‐1* in response to WCS417 (Fig. 5c). In the lignification mutant *myb15‐2*, there is increased accumulation of *p*‐coumaric acid, coumaroylagmatine, and feruloylagmatine, but decreased levels of feruloylputrescine and scopoletin (Chezem *et al*., 2017). Notably, scopoletin has been reported to play a role in the interaction between Arabidopsis and WCS417 (Stringlis *et al*., 2018b; Yu *et al*., 2021). Therefore, the altered composition or concentration of secondary metabolites in *myb15‐2* root exudates (Fig. S7) may affect WCS417 growth (Fig. 2c). Further studies should focus on the functions of identified metabolites in the interaction between Arabidopsis and WCS417.

### The interplay among defense components in plant–microbe interactions

This study revealed intricate interactions among multiple defense barriers that affect plant responses in the Arabidopsis‐WCS417 association. For example, camalexin biosynthesis‐related genes were upregulated in *myb15‐2* and *myb34/51/122* (Fig. 5c). Additionally, WCS417 induced a faster and stronger ROS burst in all the tested mutants except *bdg‐1*, including *pmr4‐1*, compared with Col‐0 (Fig. 6c,d). Previous studies reported that heat‐killed WCS417 can induce callose deposition in Arabidopsis roots, similar to the effect of flg22 (Millet *et al*., 2010); with ROS positively mediating flg22‐induced callose deposition (Luna *et al*., 2011). These findings imply that the interplay between the ROS burst and callose deposition may influence the Arabidopsis‐WCS417 association. Additionally, our RNA sequencing data revealed stronger expression of defense‐related genes in the defense barrier mutants compared with Col‐0, further emphasizing the intricate interplay among various defense barriers. These complex interactions between defense components merit further investigation to better understand their roles in plant–microbe communication.

This study highlights the importance of structural (cutin, suberin, callose, and lignin) and chemical (camalexin and glucosinolate) defense barriers in the beneficial interactions between Arabidopsis and the model rhizobacterium WCS417. An intact immune system is essential for optimal plant growth promotion, while deficiencies in certain defense barriers alter growth‐ and defense‐related processes, potentially leading to neutral or even detrimental plant–microbe interactions. Understanding how defense barriers shape beneficial plant–microbe associations could pave the way for developing microbe‐based strategies to enhance plant growth and promote sustainable agriculture.

## Competing Interests

None declared.

## Author Contributions

JZ, CMJP and IAS contributed to the conceptualization. JZ, MUA, MJJS, FW, GK and LD contributed to the methodology. JZ, MJJS, RQ, RJ, FW, GK, LD and IAS contributed to the formal analysis. JZ, MUA and IAS contributed to the investigation. JZ, and IAS contributed to the writing – original draft. JZ, MUA, RQ, RJ, CMJP and IAS contributed to the writing – review and editing. JZ, RQ and IAS contributed to the visualization. JZ and CMJP contributed to the funding acquisition.

## Disclaimer

The New Phytologist Foundation remains neutral with regard to jurisdictional claims in maps and in any institutional affiliations.

## Supporting information


**Fig. S1** Influences of *Pseudomonas simiae* WCS417 on shoot growth and root development of *Arabidopsis thaliana* Col‐0 in the *in vitro* experimental system by Herrera Paredes *et al*. (2018).
**Fig. S2** Shoot growth and root architecture changes of *Arabidopsis thaliana* Col‐0 and root defense barrier mutants in response to *Pseudomonas simiae* WCS417.
**Fig. S3** Principal component analysis (PCA) based on phenotypes of *Arabidopsis thaliana* Col‐0 and root defense barrier mutants in response to *Pseudomonas simiae* WCS417.
**Fig. S4** Effects of Milli‐Q water and *Arabidopsis thaliana* root exudates on the transcriptome of *Pseudomonas simiae* WCS417.
**Fig. S5** Experimental set‐up used to test the effect of camalexin on bacterial chemotaxis.
**Fig. S6** The impact of camalexin on swimming of *Pseudomonas simiae* WCS417, *Pseudomonas syringae* pv *tomato* DC3000, *Rhizobium* sp. YAF28, and *Bacillus subtilis* GB03.
**Fig. S7** Untargeted metabolomic analysis of root exudates from *Arabidopsis thaliana* Col‐0 and selected root defense component mutants.
**Fig. S8** Principal component analysis of gene expression profiles of indicated *Arabidopsis thaliana* genotypes after treatment of the roots with either 10 mM MgSO_4_ (a) or *Pseudomonas simiae* WCS417 (b).
**Fig. S9** UpSet plot showing differentially expressed genes (DEGs) in response to *Pseudomonas simiae* WCS417 across *Arabidopsis thaliana* genotypes.
**Fig. S10** Expression levels of all the differentially expressed genes (DEGs) in roots of different *Arabidopsis thaliana* genotypes upon the treatment with *Pseudomonas simiae* WCS417.

**Table S1** Genes detected in all the *Pseudomonas simiae* WCS417 samples with an expression of at least ten read counts in at least one sample.
**Table S2** Feature table for untargeted metabolomic analysis of root exudates from *Arabidopsis thaliana* Col‐0 and selected root defense component mutants.
**Table S3** Genes detected in all the *Arabidopsis thaliana* root samples with an expression of at least ten read counts in at least one sample.
**Table S4** *Arabidopsis thaliana* root defense barrier mutants used in this study.
**Table S5** Log2 fold changes of genes in *Pseudomonas simiae* WCS417 significantly affected (log_2_ fold change ≥1.0 or ≤ −1.0, FDR < 0.05) by different root exudates compared with Milli‐Q water treatment.
**Table S6** Gene Ontology biological process terms enriched in differentially expressed genes in *Pseudomonas simiae* WCS417 in response to different root exudates shown in Fig. 3(c).
**Table S7** Log_2_ fold changes of genes in roots of different *Arabidopsis thaliana* genotypes significantly affected (Log_2_FoldChange ≥ 1.5 or ≤ −1.5, FDR < 0.01) by *Pseudomonas simiae* WCS417 treatment.
**Table S8** Gene Ontology biological process terms enriched in differentially expressed genes in roots of different *Arabidopsis thaliana* genotypes in response to *Pseudomonas simiae* WCS417 shown in Fig. 5(c).
**Table S9** Log_2_ fold changes of bacterial motility‐related genes in *Pseudomonas simiae* WCS417 in response to root exudates of *Arabidopsis thaliana* mutants *f6′h1* and *pad3‐1*.
Please note: Wiley is not responsible for the content or functionality of any Supporting Information supplied by the authors. Any queries (other than missing material) should be directed to the *New Phytologist* Central Office.

## Data Availability

The raw RNA‐Seq read data for Arabidopsis and WCS417 are deposited in the Short Read Archive (http://www.ncbi.nlm.nih.gov/sra/; BioProject ID: PRJNA1186326 and PRJNA1185295).
