# Supplementary material for: Arabidopsis root defense barriers support beneficial interactions with rhizobacterium Pseudomonas simiae WCS417
Source: New Phytol. 2025 Sep 4;248(4):2021–39. doi: 10.1111/nph.70549 (PMC12529037; doi:10.1111/nph.70549)
Supplement: Supplementary file 1 — Fig. S1 Influences of Pseudomonas simiae WCS417 on shoot growth and root development of Arabidopsis thaliana Col‐0 in the in vitro experimental system by Herrera Paredes et al. (2018). Fig. S2 Shoot growth and root architecture changes of Arabidopsis thaliana Col‐0 and root defense barrier mutants in response to Pseudomonas simiae WCS417. Fig. S3 Principal component analysis (PCA) based on phenotypes of Arabidopsis thaliana Col‐0 and root defense barrier mutants in response to Pseudomonas simiae WCS417. Fig. S4 Effects of Milli‐Q water and Arabidopsis thaliana root exudates on the transcriptome of Pseudomonas simiae WCS417. Fig. S5 Experimental set‐up used to test the effect of camalexin on bacterial chemotaxis. Fig. S6 The impact of camalexin on swimming of Pseudomonas simiae WCS417, Pseudomonas syringae pv tomato DC3000, Rhizobium sp. YAF28, and Bacillus subtilis GB03. Fig. S7 Untargeted metabolomic analysis of root exudates from Arabidopsis thaliana Col‐0 and selected root defense component mutants. Fig. S8 Principal component analysis of gene expression profiles of indicated Arabidopsis thaliana genotypes after treatment of the roots with either 10 mM MgSO4 (a) or Pseudomonas simiae WCS417 (b). Fig. S9 UpSet plot showing differentially expressed genes (DEGs) in response to Pseudomonas simiae WCS417 across Arabidopsis thaliana genotypes. Fig. S10 Expression levels of all the differentially expressed genes (DEGs) in roots of different Arabidopsis thaliana genotypes upon the treatment with Pseudomonas simiae WCS417. [file NPH-248-2021-s001.pdf]

New Phytologist **Supporting Information**

Article title: **Arabidopsis root defense barriers support beneficial interactions with rhizobacterium *Pseudomonas simiae* WCS417**

Authors: Jiayu Zhou [1, 2], Melissa Uribe Acosta [1], Max J. J. Stassen [1], Run Qi [1], Ronnie de Jonge [1, 3], Fred White [4], Gertjan Kramer [4], Lemeng Dong [4], Corné M.J. Pieterse [1] and Ioannis A. Stringlis [1, 5*]

Article acceptance date: 14 August 2025

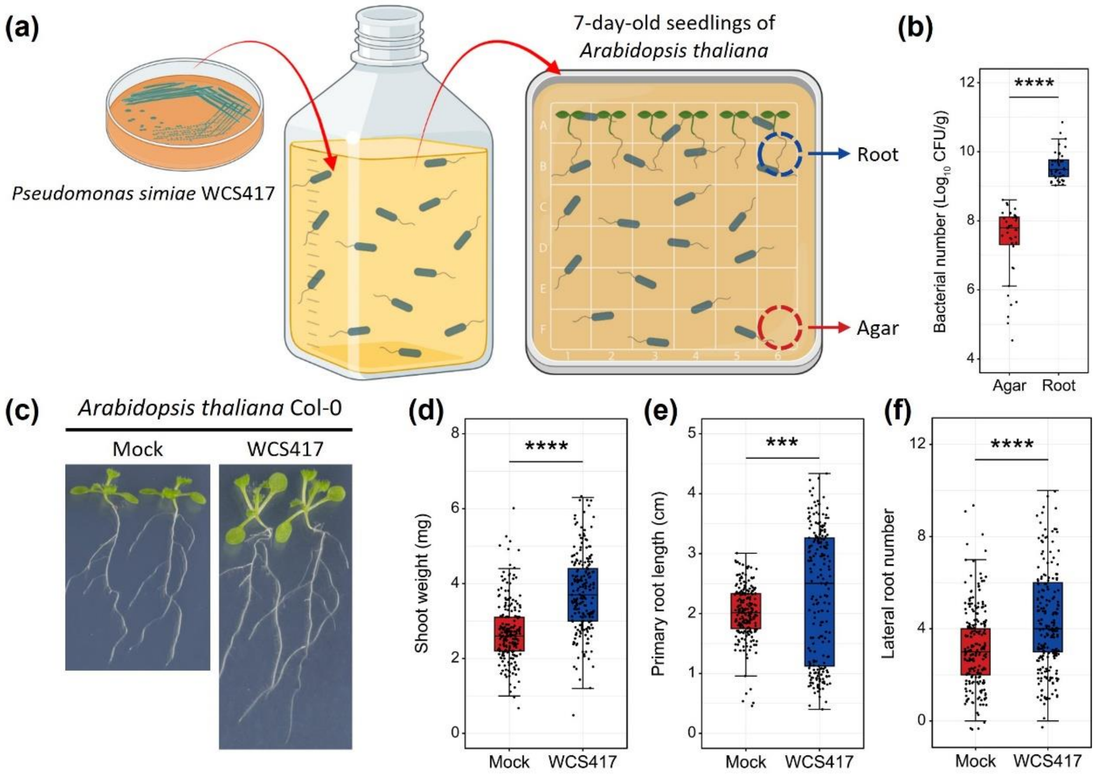

Supplementary Figure S1. Influences of *Pseudomonas simiae* WCS417 on shoot growth and root development of *Arabidopsis thaliana* Col-0 in the *in vitro* experimental system by Herrera Paredes *et al* (2018). **(a)** Schematic diagram of the experimental system in which WCS417 was mixed into Hoagland medium without sucrose at a final concentration of 10^5 CFU/mL, allowing it to colonize developing Arabidopsis roots growing on the agar surface. **(b)** Bacterial CFUs retrieved from the agar away from roots indicated as Agar in (a), and on the root surface indicated as Root in (a) at 7 days after transplanting of seedlings to Hoagland medium without sucrose containing 10^5 CFU/mL WCS417. Representative photos **(c)**, shoot weight **(d)**, primary root length **(e)**, and lateral root number **(f)** of Col-0 at 7 days after transplanting of seedlings to Hoagland medium without sucrose containing 10^5 CFU/mL WCS417 or an equal volume of 10 mM $MgSO_4$ (Mock). In the boxplots, the horizontal line inside each box represents the median; the top and bottom edges of each box represent the 75th and 25th quartiles, respectively; and the upper and lower whiskers extend to 1.5× the interquartile range from the top and bottom of the box, respectively. Asterisks represent significant differences between different treatments (Student's *t*-test; *** $P < 0.001$, **** $P < 0.0001$). In the case of root colonization, data points represent either agar samples (n = 39) or root samples (n = 41) with each point corresponding to a biological replicate. In the case of morphological characters, each data point represents a single Arabidopsis seedling, also treated as a biological replicate. The sample sizes are n = 208 for Mock and n = 209 for WCS417. Top part of Figure was created in BioRender. Stringlis, I. (2025) https://BioRender.com/rmhiajx

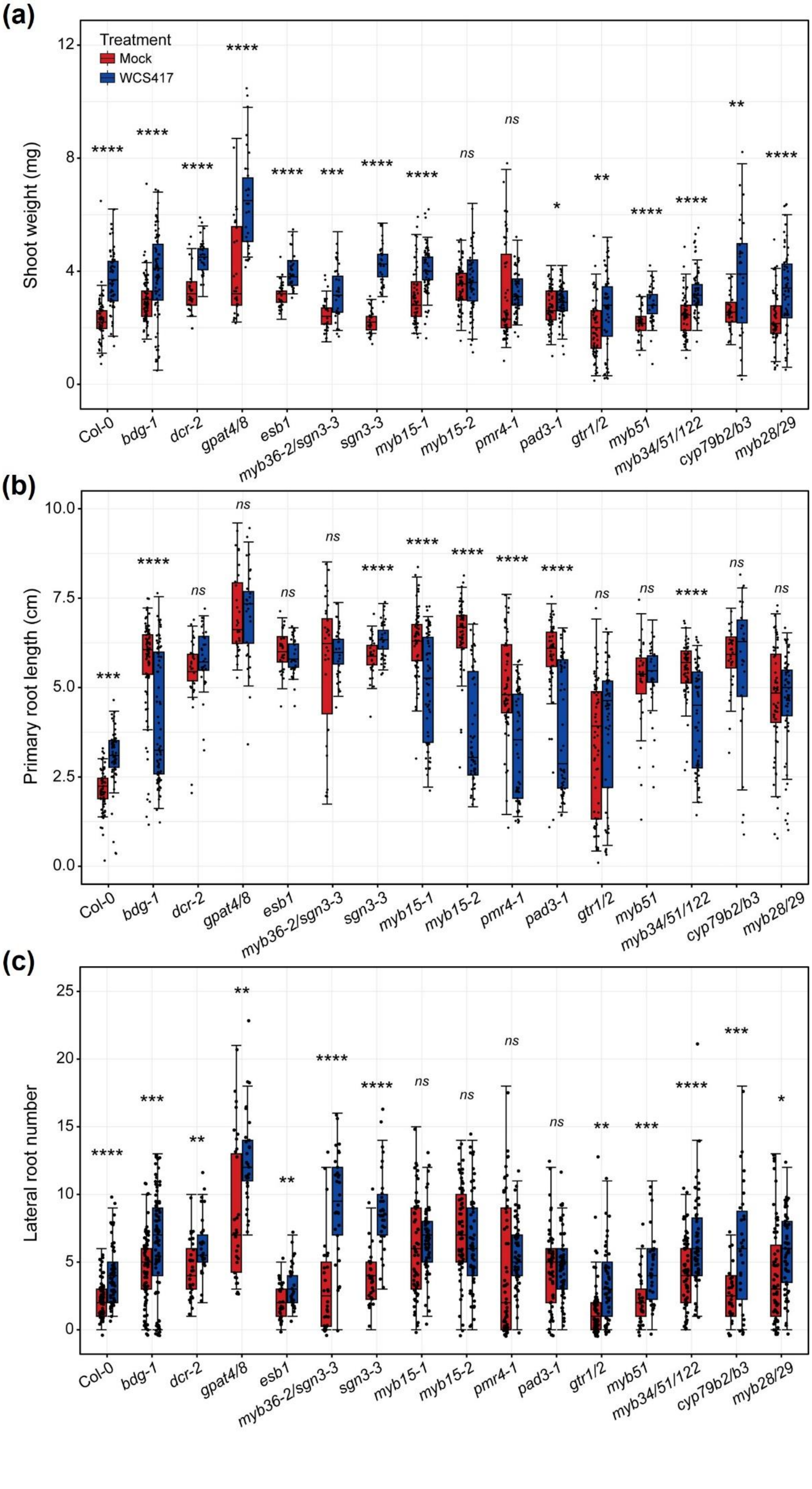
(a)
Treatment
Mock
WCS417
Shoot weight (mg)
0
4
8
12
****
****
****
****
****
***
****
****
ns
ns
*
**
****
****
**
****
Col-0
bdg-1
dcr-2
gpat4/8
esb1
myb36-2/sgn3-3
sgn3-3
myb15-1
myb15-2
pmr4-1
pad3-1
gtr1/2
myb51
myb34/51/122
cyp79b2/b3
myb28/29
(b)
Primary root length (cm)
0.0
2.5
5.0
7.5
10.0
***
****
ns
ns
ns
ns
****
****
****
****
****
ns
ns
****
ns
ns
Col-0
bdg-1
dcr-2
gpat4/8
esb1
myb36-2/sgn3-3
sgn3-3
myb15-1
myb15-2
pmr4-1
pad3-1
gtr1/2
myb51
myb34/51/122
cyp79b2/b3
myb28/29
(c)
Lateral root number
0
5
10
15
20
25
****
***
**
**
**
****
****
ns
ns
ns
ns
**
***
****
***
*
Col-0
bdg-1
dcr-2
gpat4/8
esb1
myb36-2/sgn3-3
sgn3-3
myb15-1
myb15-2
pmr4-1
pad3-1
gtr1/2
myb51
myb34/51/122
cyp79b2/b3
myb28/29

Supplementary Figure S2. Shoot growth and root architecture changes of *Arabidopsis thaliana* Col-0 and root defense barrier mutants in response to *Pseudomonas simiae* WCS417. Shoot fresh weight **(a)**, primary root length **(b)** and lateral root number **(c)** of Col-0 and different mutants at 7 days after transplanting seedlings to Hoagland medium without sucrose containing 10^5 CFU/mL WCS417 or an equal volume of 10 mM $MgSO_4$ (Mock). In the boxplots, the horizontal line inside each box represents the median; the top and bottom edges of each box represent the 75th and 25th quartiles, respectively; and the upper and lower whiskers extend to 1.5× the interquartile range from the top and bottom of the box, respectively. Asterisks represent statistically significant differences between different treatments (Mock and WCS417) in each genotype (Student's *t*-test; * $P < 0.05$, ** $P < 0.01$, *** $P < 0.001$, **** $P < 0.0001$; *ns*, not significant). Each data point is a biological replicate, representing a single Arabidopsis seedling. The number of seedlings per genotype and treatment is as follows: Col-0 (Mock: n = 60; WCS417: n = 59), *bdg-1* (Mock: n = 80; WCS417: n = 80), *dcr-2* (Mock: n = 30; WCS417: n = 30), *gpat4/8* (Mock: n = 30; WCS417: n = 30), *esb1* (Mock: n = 30; WCS417: n = 30), *myb36-2/sgn3-3* (Mock: n = 30; WCS417: n = 30), *sgn3-3* (Mock: n = 30; WCS417: n = 30), *myb15-1* (Mock: n = 60; WCS417: n = 60), *myb15-2* (Mock: n = 60; WCS417: n = 60), *pmr4-1* (Mock: n = 60; WCS417: n = 60), *pad3-1* (Mock: n = 60; WCS417: n = 60), *gtr1/2* (Mock: n = 60; WCS417: n = 55), *myb51* (Mock: n = 30; WCS417: n = 30), *myb34/51/122* (Mock: n = 60; WCS417: n = 60), *cyp79b2/b3* (Mock: n = 30; WCS417: n = 30), and *myb28/29* (Mock: n = 60; WCS417: n = 59).

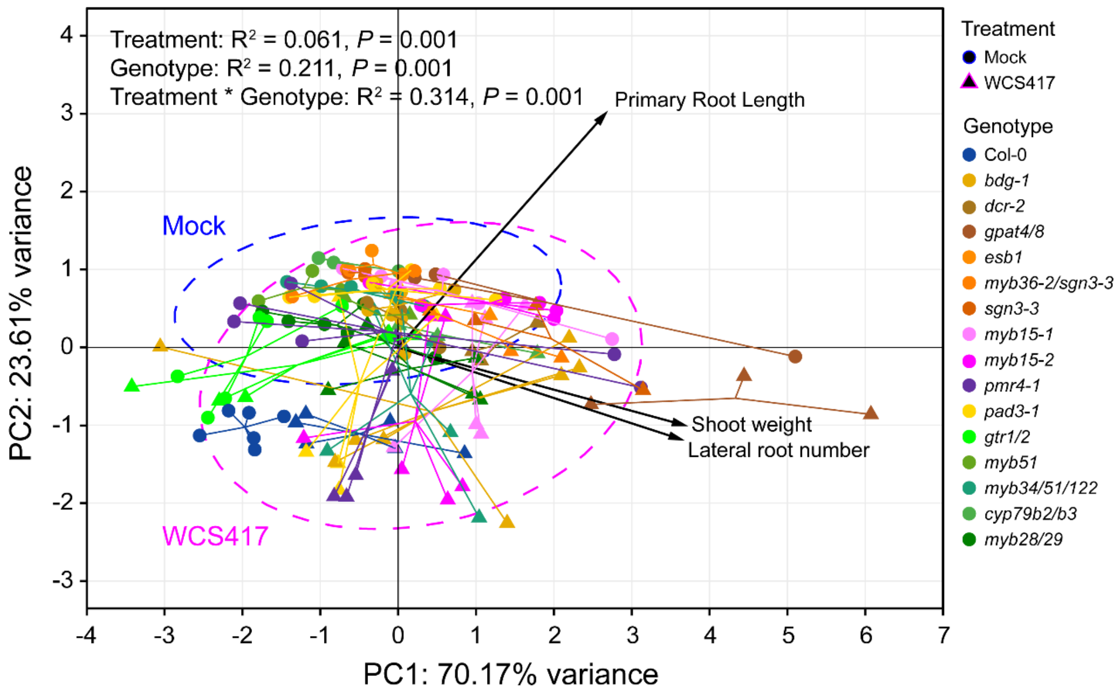

Supplementary Figure S3. Principal component analysis (PCA) based on phenotypes of *Arabidopsis thaliana* Col-0 and root defense barrier mutants in response to *Pseudomonas simiae* WCS417. PCA was performed based on shoot fresh weight, primary root length and lateral root number. Each data point in the PCA plot represents the average value of all the seedlings from one plate. The number of plates per genotype and treatment is as follows: Col-0 (Mock: *n* = 6; WCS417: *n* = 6), *bdg-1* (Mock: *n* = 8; WCS417: *n* = 8), *dcr-2* (Mock: *n* = 3; WCS417: *n* = 3), *gpat4/8* (Mock: *n* = 3; WCS417: *n* = 3), *esb1* (Mock: *n* = 3; WCS417: *n* = 3), *myb36-2/sgn3-3* (Mock: *n* = 3; WCS417: *n* = 3), *sgn3-3* (Mock: *n* = 3; WCS417: *n* = 3), *myb15-1* (Mock: *n* = 6; WCS417: *n* = 6), *myb15-2* (Mock: *n* = 6; WCS417: *n* = 6), *pmr4-1* (Mock: *n* = 6; WCS417: *n* = 6), *pad3-1* (Mock: *n* = 6; WCS417: *n* = 6), *gtr1/2* (Mock: *n* = 6; WCS417: *n* = 6), *myb51* (Mock: *n* = 3; WCS417: *n* = 3), *myb34/51/122* (Mock: *n* = 6; WCS417: *n* = 6), *cyp79b2/b3* (Mock: *n* = 3; WCS417: *n* = 3), and *myb28/29* (Mock: *n* = 6; WCS417: *n* = 6). To quantify the contribution of treatments and genotypes, permutational multivariate analysis of variance (PERMANOVA) was performed based on individual seedling data. The number of seedlings per genotype and treatment is as follows: Col-0 (Mock: *n* = 60; WCS417: *n* = 59), *bdg-1* (Mock: *n* = 80; WCS417: *n* = 80), *dcr-2* (Mock: *n* = 30; WCS417: *n* = 30), *gpat4/8* (Mock: *n* = 30; WCS417: *n* = 30), *esb1* (Mock: *n* = 30; WCS417: *n* = 30), *myb36-2/sgn3-3* (Mock: *n* = 30; WCS417: *n* = 30), *sgn3-3* (Mock: *n* = 30; WCS417: *n* = 30), *myb15-1* (Mock: *n* = 60; WCS417: *n* = 60), *myb15-2* (Mock: *n* = 60; WCS417: *n* = 60), *pmr4-1* (Mock: *n* = 60; WCS417: *n* = 60), *pad3-1* (Mock: *n* = 60; WCS417: *n* = 60), *gtr1/2* (Mock: *n* = 60; WCS417: *n* = 55), *myb51* (Mock: *n* = 30; WCS417: *n* = 30), *myb34/51/122* (Mock: *n* = 60; WCS417: *n* = 60), *cyp79b2/b3* (Mock: *n* = 30; WCS417: *n* = 30), and *myb28/29* (Mock: *n* = 60; WCS417: *n* = 59).

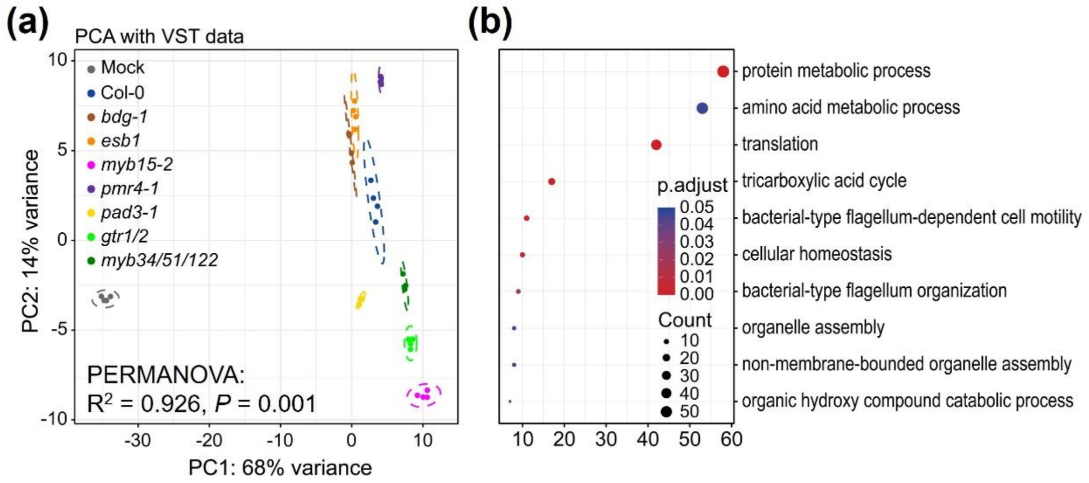

Supplementary Figure S4. Effects of Milli-Q water and *Arabidopsis thaliana* root exudates on the transcriptome of *Pseudomonas simiae* WCS417. (a) Principal component analysis (PCA) of WCS417 transcriptional profiles in response to Milli-Q water and Arabidopsis root exudates. Each color represents bacterial samples (n = 4) treated with Milli-Q water (Mock) or root exudates from the indicated Arabidopsis genotypes. Ellipses correspond to t-distributions fitted to each treatment (95% confidence interval). **(b)** Bubble plot showing Gene Ontology (GO) terms enriched in genes responsible for the separation in PC1 component in PCA plot shown in (a). Dot color represents adjusted P values calculated using a hypergeometric test, with red indicating lower and blue indicating higher values. Dot size represents the count of genes enriched in each GO term, with larger ones indicating more genes and smaller ones indicating fewer genes.

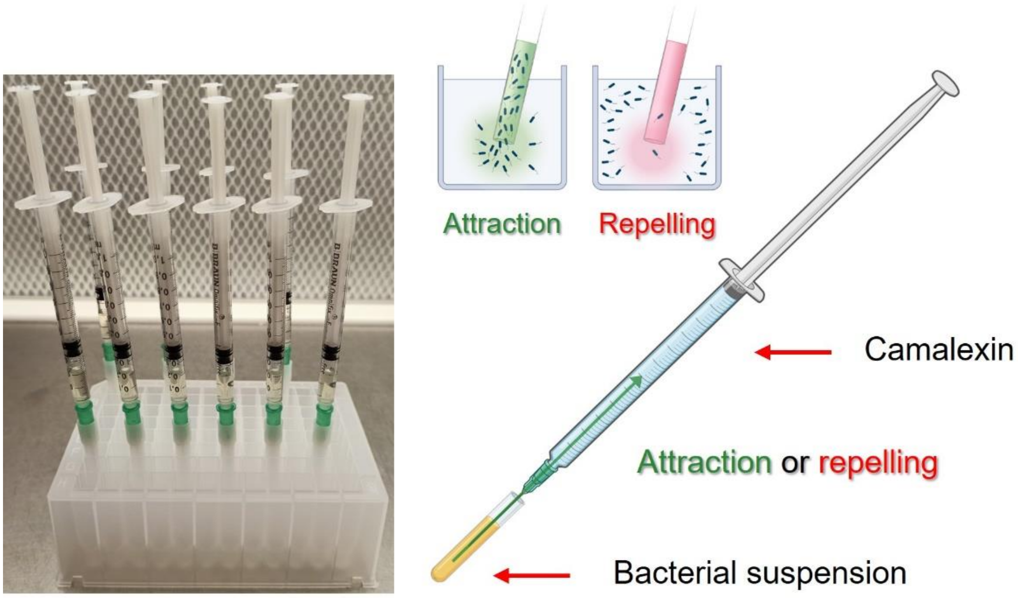

Supplementary Figure S5. Experimental set-up used to test the effect of camalexin on bacterial chemotaxis. A volume of 200 µL of 1.0 mM camalexin in 1% DMSO, 10 mM $MgSO_4$ or 1% DMSO, 10 mM $MgSO_4$ as a control was drawn into a syringe with a needle. The needle was placed in a 250-µL WCS417 suspension (OD600 = 0.1) and the number of bacterial cells moving into the syringe containing camalexin or not was measured and compared to evaluate attraction or repelling effects of camalexin on WCS417. Right panel was created in BioRender. Stringlis, I. (2025) https://BioRender.com/f2bbouo

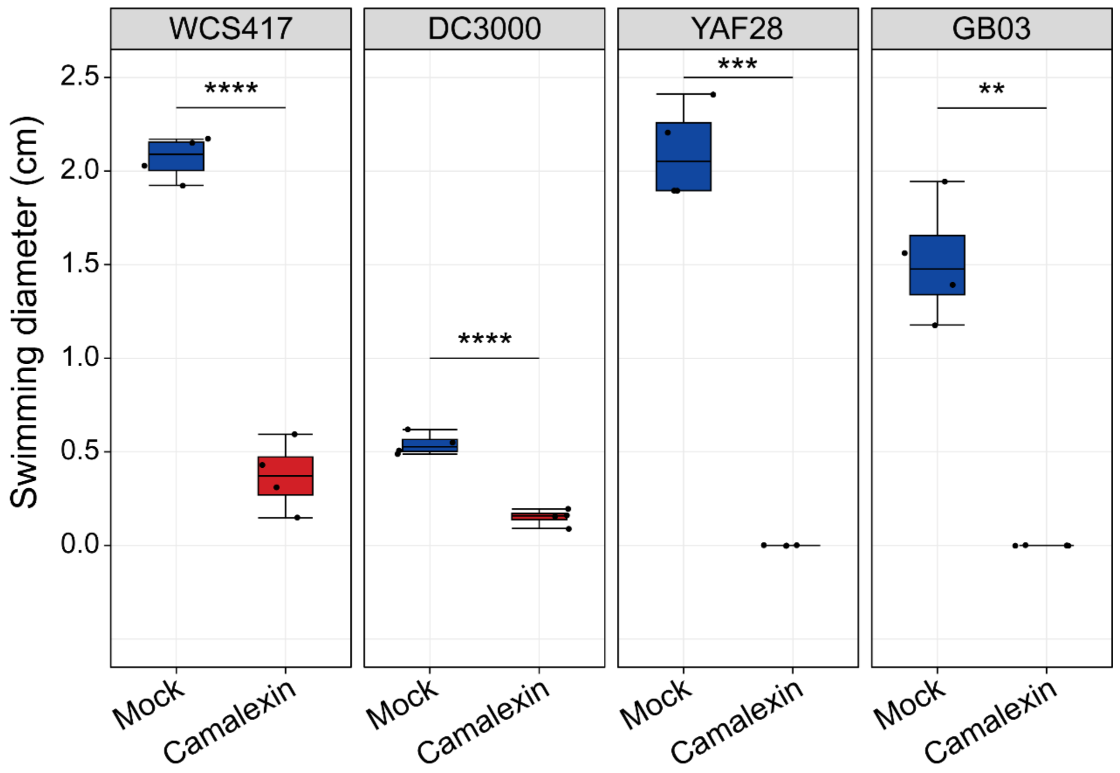

Supplementary Figure S6. The impact of camalexin on swimming of *Pseudomonas simiae* WCS417, *Pseudomonas syringae* pv. *tomato* DC3000, *Rhizobium* sp. YAF28, and *Bacillus subtilis* GB03. In the boxplots, the horizontal line inside each box represents the median; the top and bottom edges of each box represent the 75th and 25th quartiles, respectively; and the upper and lower whiskers extend to 1.5 × the interquartile range from the top and bottom of the box, respectively. Asterisks represent statistically significant differences between swimming diameter of each strain after 20 hours of growth on semi-solidified medium supplemented with DMSO (Mock) and 1 mM camalexin (Student's *t*-test; ** $P < 0.01$, *** $P < 0.001$, $n = 4$). Each data point represents a biological replicate.

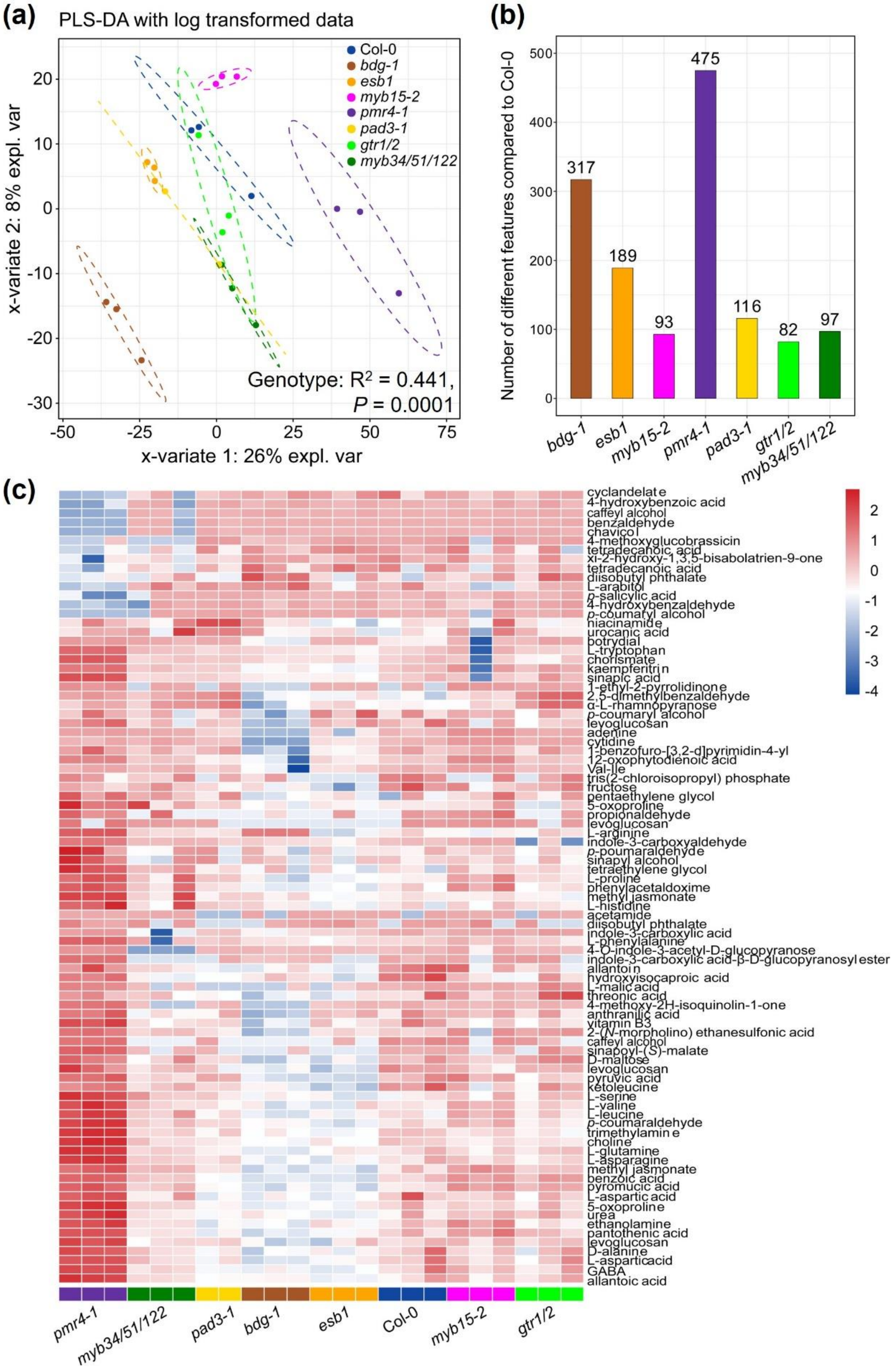

(a)
PLS-DA with log transformed data
Col-0
bdg-1
esb1
myb15-2
pmr4-1
pad3-1
gtr1/2
myb34/51/122
Genotype: R2 = 0.441,
P = 0.0001
x-variate 1: 26% expl. var
x-variate 2: 8% expl. var
-50
-25
0
25
50
75
20
10
0
-10
-20
-30
(b)
Number of different features compared to Col-0
317
189
93
475
116
82
97
bdg-1
esb1
myb15-2
pmr4-1
pad3-1
gtr1/2
myb34/51/122
500
400
300
200
100
0
(c)
cyclandelate
4-hydroxybenzoic acid
caffeyl alcohol
benzaldehyde
chavicol
4-methoxyglucobrassicin
tetradecanoic acid
xi-2-hydroxy-1,3,5-bisabolatrien-9-one
tetradecanoic acid
diisobutyl phthalate
L-arabitol
p-salicylic acid
4-hydroxybenzaldehyde
p-coumaryl alcohol
niacinamide
urocanic acid
botrydial
L-tryptophan
chorismate
kaempferitrin
sinapic acid
1-ethyl-2-pyrrolidinone
2,5-dimethylbenzaldehyde
α-L-rhamnopyranose
p-coumaryl alcohol
levoglucosan
adenine
cytidine
1-benzofuro-[3,2-d]pyrimidin-4-yl
12-oxophytodienoic acid
Val-Ile
tris(2-chloroisopropyl) phosphate
fructose
pentaethylene glycol
5-oxoproline
propionaldehyde
levoglucosan
L-arginine
indole-3-carboxyaldehyde
p-coumaraldehyde
sinapyl alcohol
tetraethylene glycol
L-proline
phenylacetaldoxime
methyl jasmonate
L-histidine
acetamide
diisobutyl phthalate
indole-3-carboxylic acid
L-phenylalanine
4-O-indole-3-acetyl-D-glucopyranose
indole-3-carboxylic acid-β-D-glucopyranosyl ester
allantoin
hydroxyisocaproic acid
L-malic acid
threonic acid
4-methoxy-2H-isoquinolin-1-one
anthranilic acid
vitamin B3
2-(N-morpholino) ethanesulfonic acid
caffeyl alcohol
sinapoyl-(S)-malate
D-maltose
levoglucosan
pyruvic acid
ketoleucine
L-serine
L-valine
L-leucine
p-coumaraldehyde
trimethylamine
choline
L-glutamine
L-asparagine
methyl jasmonate
benzoic acid
pyromucic acid
L-aspartic acid
5-oxoproline
urea
ethanolamine
pantothenic acid
levoglucosan
D-alanine
L-asparticacid
GABA
allantoic acid
2
1
0
-1
-2
-3
-4
pmr4-1
myb34/51/122
pad3-1
bdg-1
esb1
Col-0
myb15-2
gtr1/2

Supplementary Figure S7. Untargeted metabolomic analysis of root exudates from *Arabidopsis thaliana* Col-0 and selected root defense component mutants. (a) Partial least squares discriminant analysis (PLS-DA) plot shows separation of different root exudate metabolome. Colors indicate different genotypes; ellipses correspond to *t*-distributions fitted to each treatment (95% confidence interval). **(b)** Number of features with significantly different intensities in root exudates of selected mutants compared to Col-0. **(c)** Heatmap of annotated features across genotypes, with lower intensities in blue and higher intensities in red.

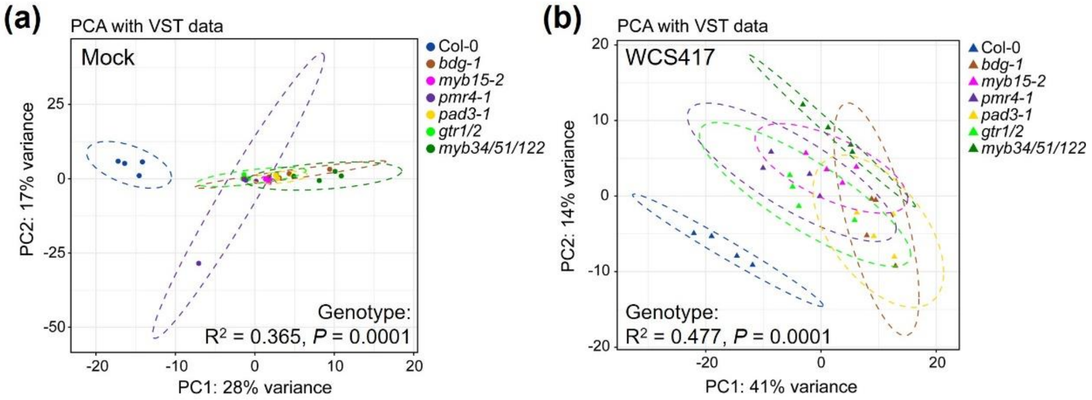

Supplementary Figure S8. Principal component analysis of gene expression profiles of indicated *Arabidopsis thaliana* genotypes after treatment of the roots with either 10 mM $MgSO_4$ (a) or *Pseudomonas simiae* WCS417 (b). Different colors represent different genotypes and different shapes represent 10 mM $MgSO_4$ (Mock) or WCS417 treated samples (n = 4). Ellipses correspond to t-distributions fitted to each treatment (95% confidence interval).

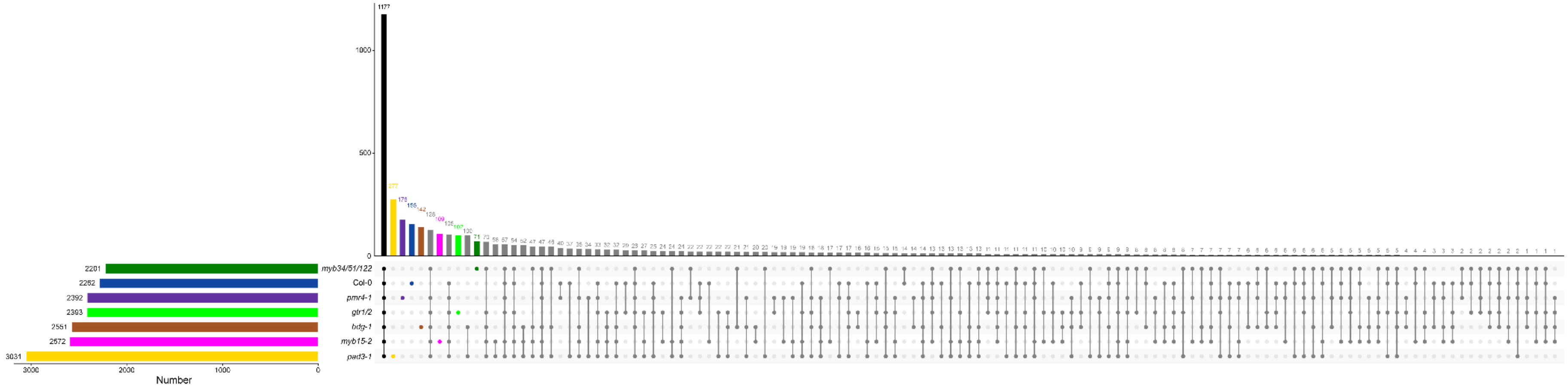

1177
1000
500
0
277
175
155
142
128
109
125
107
100
71
myb34/51/122
Col-0
pmr4-1
gtr1/2
bdg-1
myb15-2
pad3-1
2201
2262
2392
2393
2551
2572
3031
3000
2000
1000
0
Number

Supplementary Figure S9 UpSet plot showing differentially expressed genes (DEGs) in response to *Pseudomonas simiae* WCS417 across *Arabidopsis thaliana* genotypes. DEGs were identified by comparing WCS417- and mock-treated seedlings within each genotype based on four biological replicates ($\log_2$ fold change $\geqslant$ 1.5 or $\leqslant$ -1.5, FDR < 0.01). The plot highlights both DEGs shared across all the genotypes and genotype-unique DEGs with different colors indicating individual genotypes.

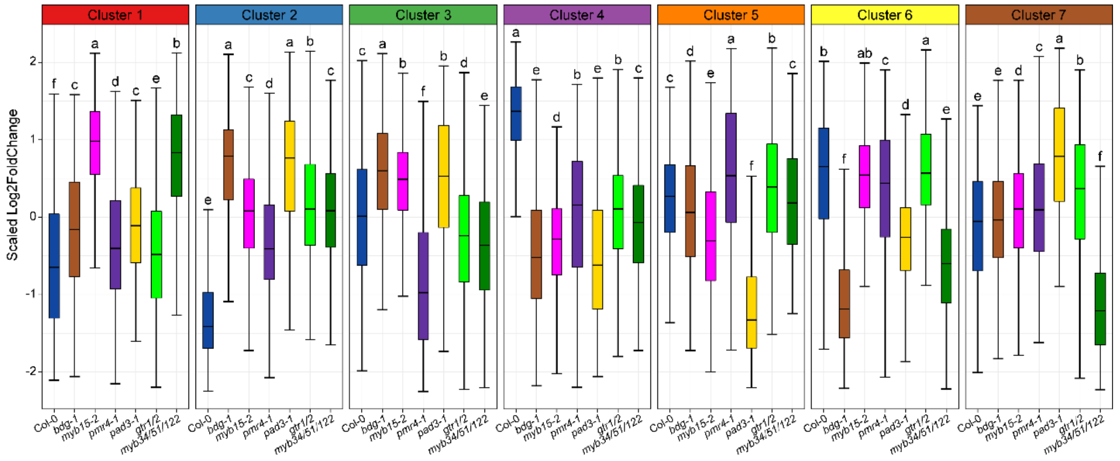

Supplementary Figure S10. Expression levels of all the differentially expressed genes (DEGs) in roots of different *Arabidopsis thaliana* genotypes upon the treatment with *Pseudomonas simiae* WCS417. DEGs are divided into 7 clusters by *k*-means, which is consistent with the clusters of the heatmap in Fig. 5C. Data are scaled $\log_2$-transformed fold changes. In the boxplots, the horizontal line inside each box represents the median; the top and bottom edges of each box represent the 75th and 25th quartiles, respectively; and the upper and lower whiskers extend to 1.5 × the interquartile range from the top and bottom of the box, respectively. Different lowercase letters represent significant differences among indicated genotypes (One-way ANOVA with least significant difference test, $P < 0.05$, $n = 10$).
